# Supplementary material for: Effective combinatorial immunotherapy for penile squamous cell carcinoma
Source: Nat Commun. 2020 May 1;11:2124. doi: 10.1038/s41467-020-15980-9 (PMC7195486; doi:10.1038/s41467-020-15980-9)
Supplement: Supplementary file 1 — Supplementary Information [file 41467_2020_15980_MOESM1_ESM.pdf]

SUPPLEMENTARY INFORMATION

Effective Combinatorial Immunotherapy for Penile Squamous Cell Carcinoma

Tianhe Huang, Xi Cheng, Jad Chahoud, Ahmed Sarhan, Pheroze Tamboli, Priya Rao, Ming Guo, Ganiraju Manyam, Li Zhang, Yu Xiang, Leng Han, Xiaoying Shang, Pingna Deng, Yanting Luo, Xuemin Lu, Shan Feng, Magaly Martinez Ferrer, Y. Alan Wang, Ronald A. DePinho, Curtis A. Pettaway, Xin Lu

Supplementary Figure 1 ~ 7

Supplementary Table 1 ~ 13

SUPPLEMENTAL FIGURES

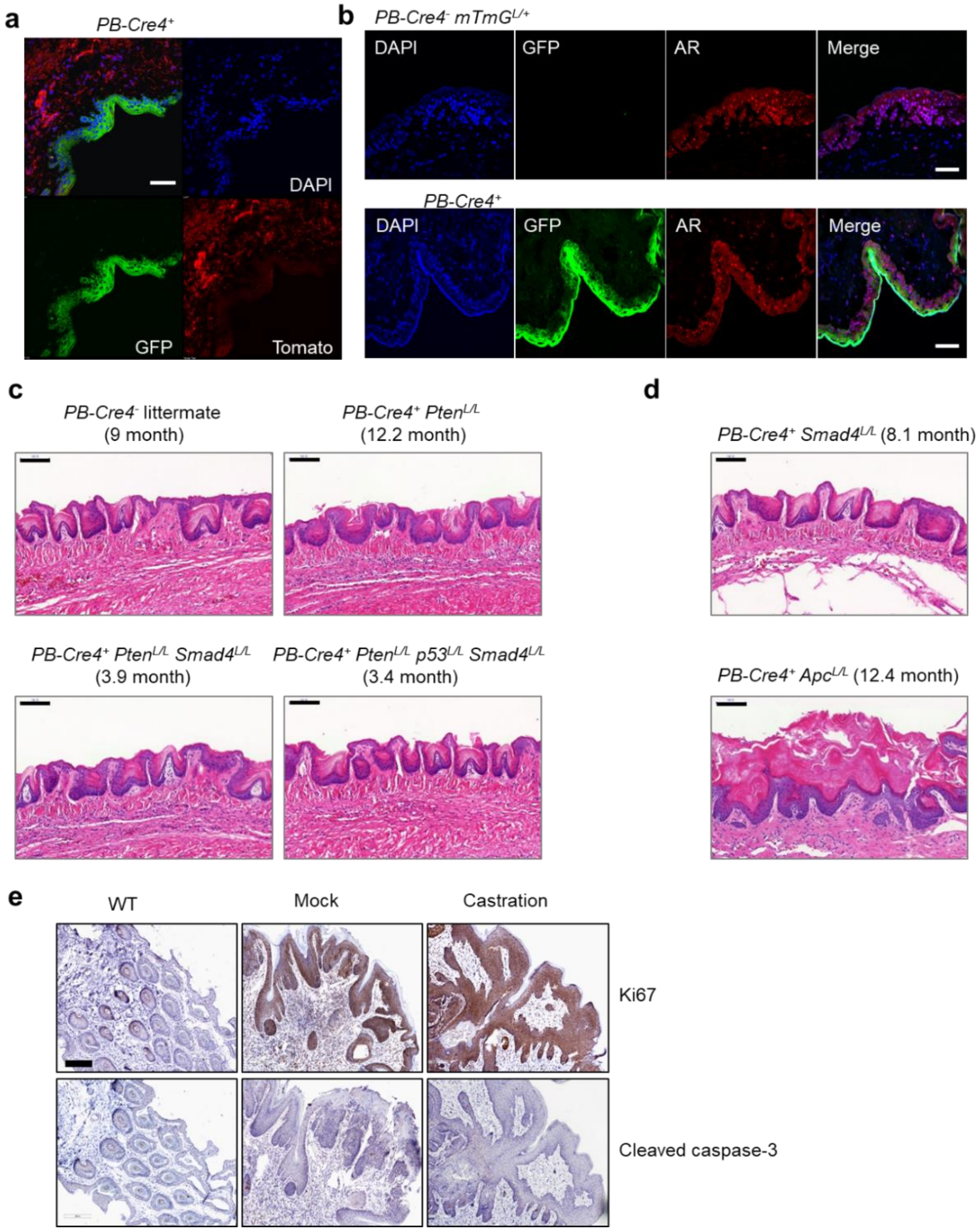

Supplementary Figure 1. Ar expression in mouse penis and penile histology of mouse models. **(a)** GFP signals in penile epithelium because of Cre-mediated recombination of the mTmG allele in *PB-Cre4*$^{+}$ *mTmG*$^{L/+}$ mice, detected by fluorescence imaging of freshly sectioned mouse penis. Scale bar 50 µm. **(b)** Nuclear Ar expression in GFP^{+} penile squamous epithelial cells in *PB-Cre4*$^{+}$ *mTmG*$^{L/+}$ mice, detected by fluorescence co-staining and confocal microscopy. Scale bar 50 µm. **(c-d)** H&E stain of the penile epithelium of mice with indicated genotypes and ages. Scale bar 100 µm. **(e)** Representative IHC images for Ki67 and cleaved caspase-3 for the penile tumors from SA males 4 weeks post-surgery or WT control (n = 6). Scale bar 200 µm.

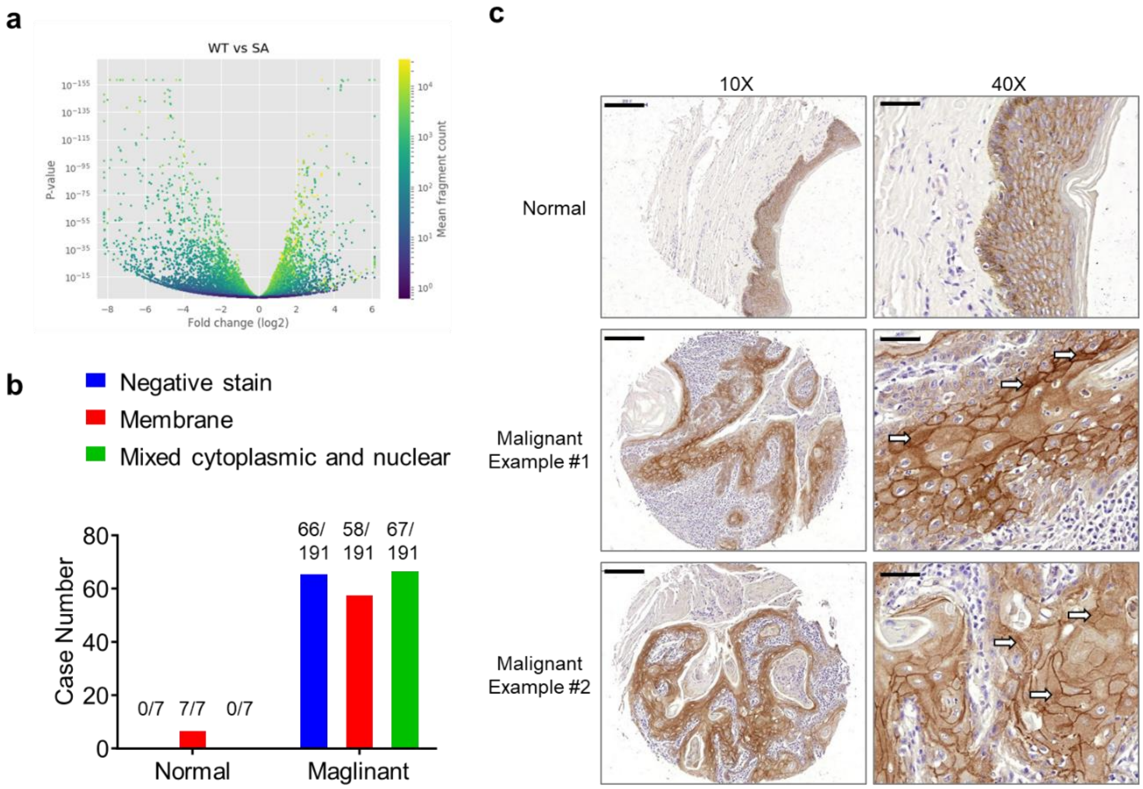

Supplementary Figure 2. Differential gene expression in mouse PSCC and β-catenin expression in human PSCC. **(a)** Volcano plot showing significantly up- and down-regulated genes in SA tumors compared with WT penises. **(b-c)** Quantification and representative images of β-catenin IHC stain of two combined tissue arrays of penile cancer, PE241 (normal 4, PSCC 20) and PE2081 (normal 3, PSCC 174). Both tissue arrays were purchased from US Biomax, Inc. Arrows indicate mixed cytoplasmic and nuclear localization of β-catenin. Scale bars 200 µm (for 10X) and 50 µm (for 40X).

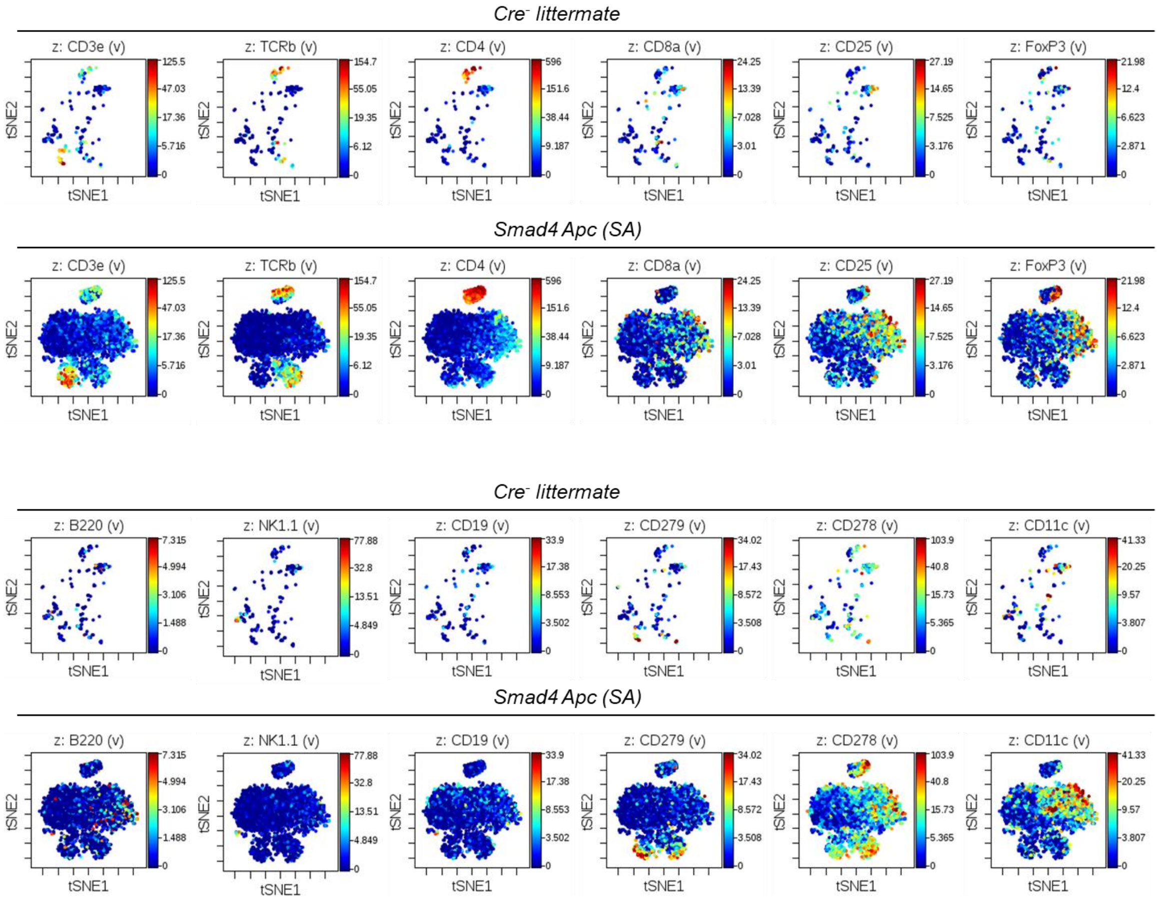

Supplementary Figure 3. Representative viSNE plots for normal and cancerous mouse penis. Dot plots are colored by the intensity of the channels shown on the top of each plot.

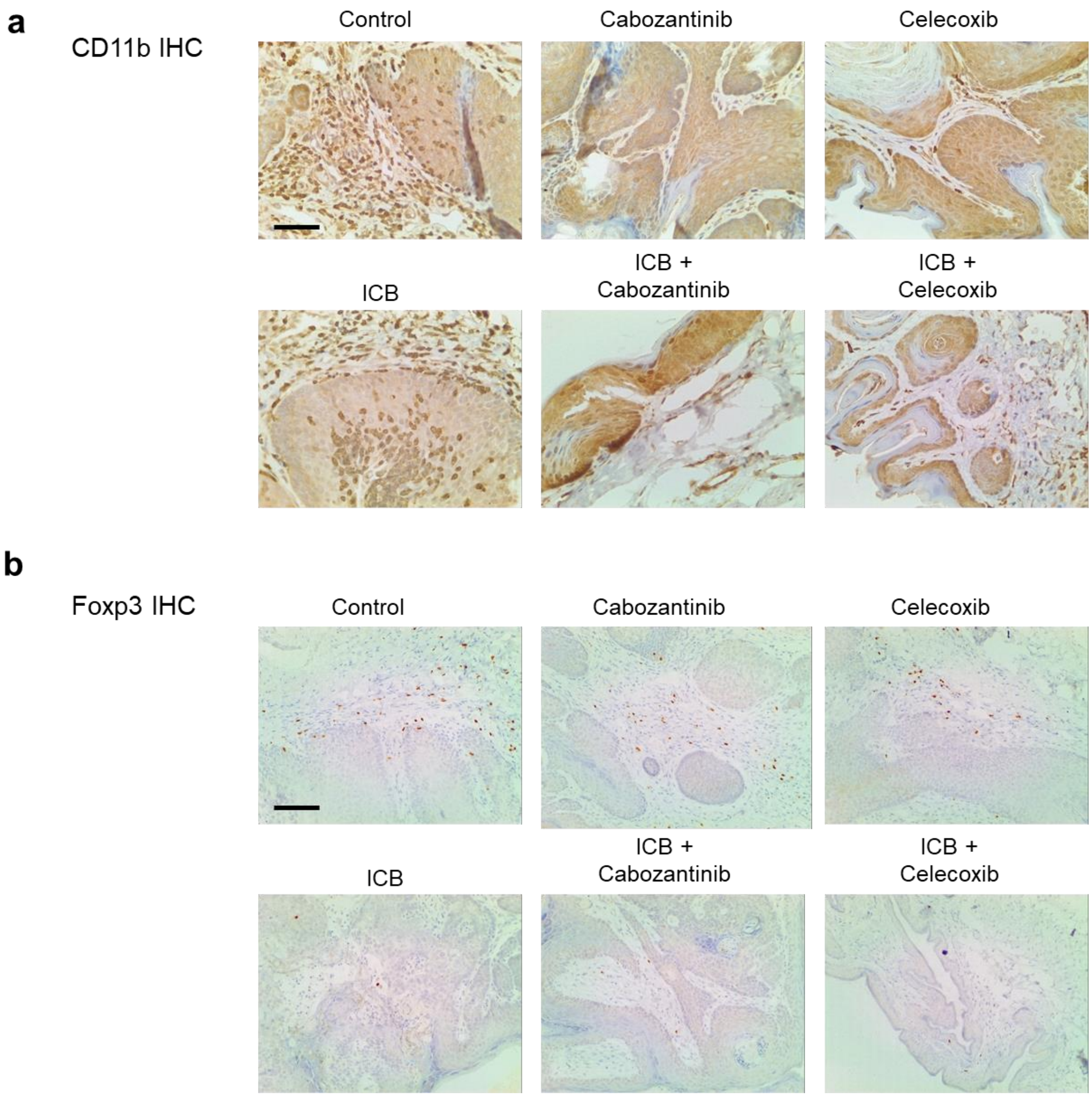

Supplementary Figure 4. Representative IHC images for combination therapy. **(a)** Representative IHC images of CD11b. Scale bar 50 μm. **(b)** Representative IHC images of Foxp3. Scale bar 100 μm.

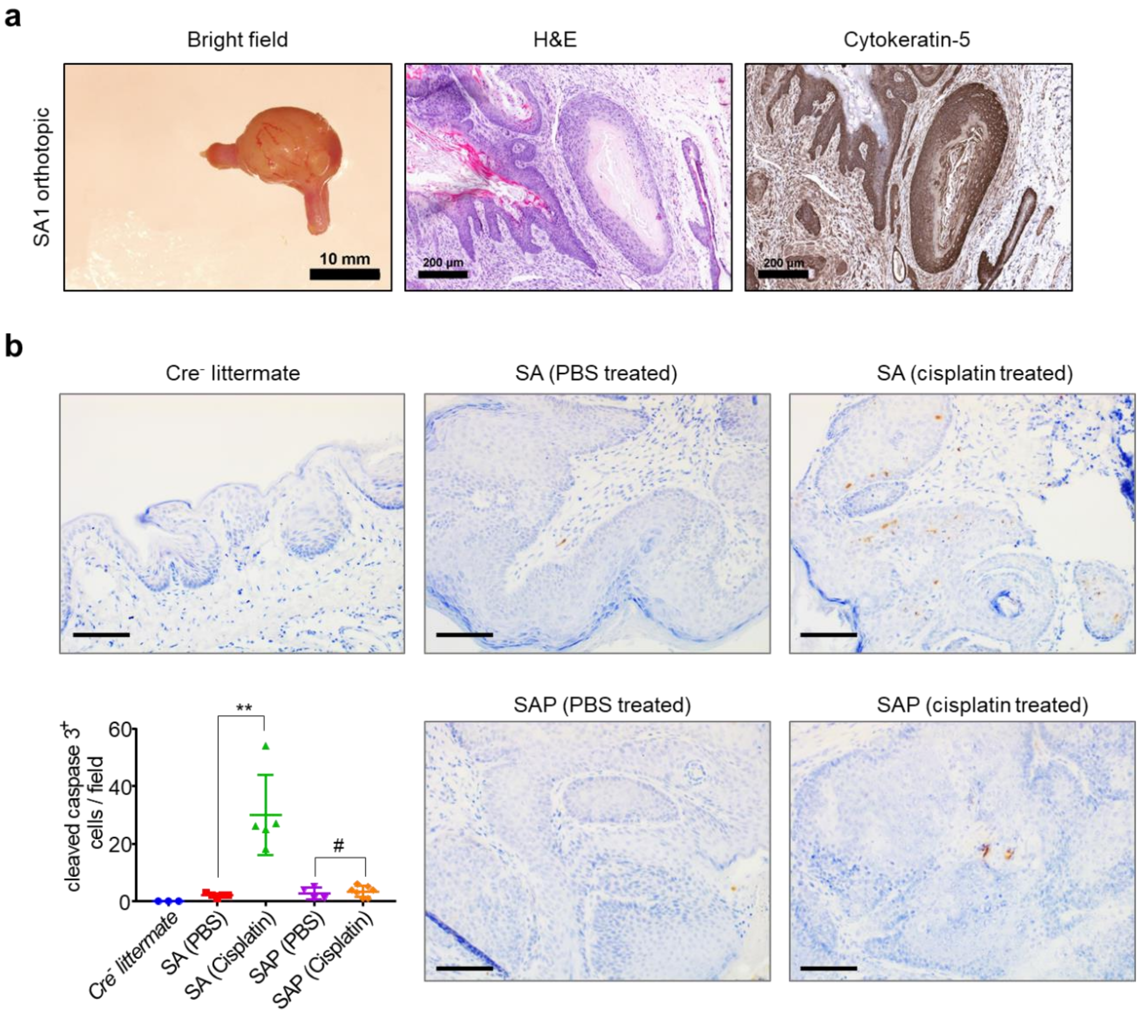

Supplementary Figure 5. Distinct apoptosis phenotype of SA and SAP tumors in response to cisplatin. **(a)** Representative penile tumor formed at Day60 after penile injection of SA1 cells into 6-week *Rag1*$^{-/-}$ males (n = 5) with H&E staining and IHC of cytokeratin-5. Scale bar 10 mm and 200 µm. **(b)** Representative IHC images and quantification of cleaved caspase-3-positive apoptotic cells per field of PBS treated or cisplatin treated penile tumors. Tumors were harvested at the endpoint of the cisplatin experiment from SA or SAP tumors (n = 3, 5, 5, 4, and 7 independent IHC images for the groups in order). Scale bar 100 µm. Data represent mean ± SD. $^{\#}P>0.05$, $^{**}P<0.01$, two-sided Student's *t* test.

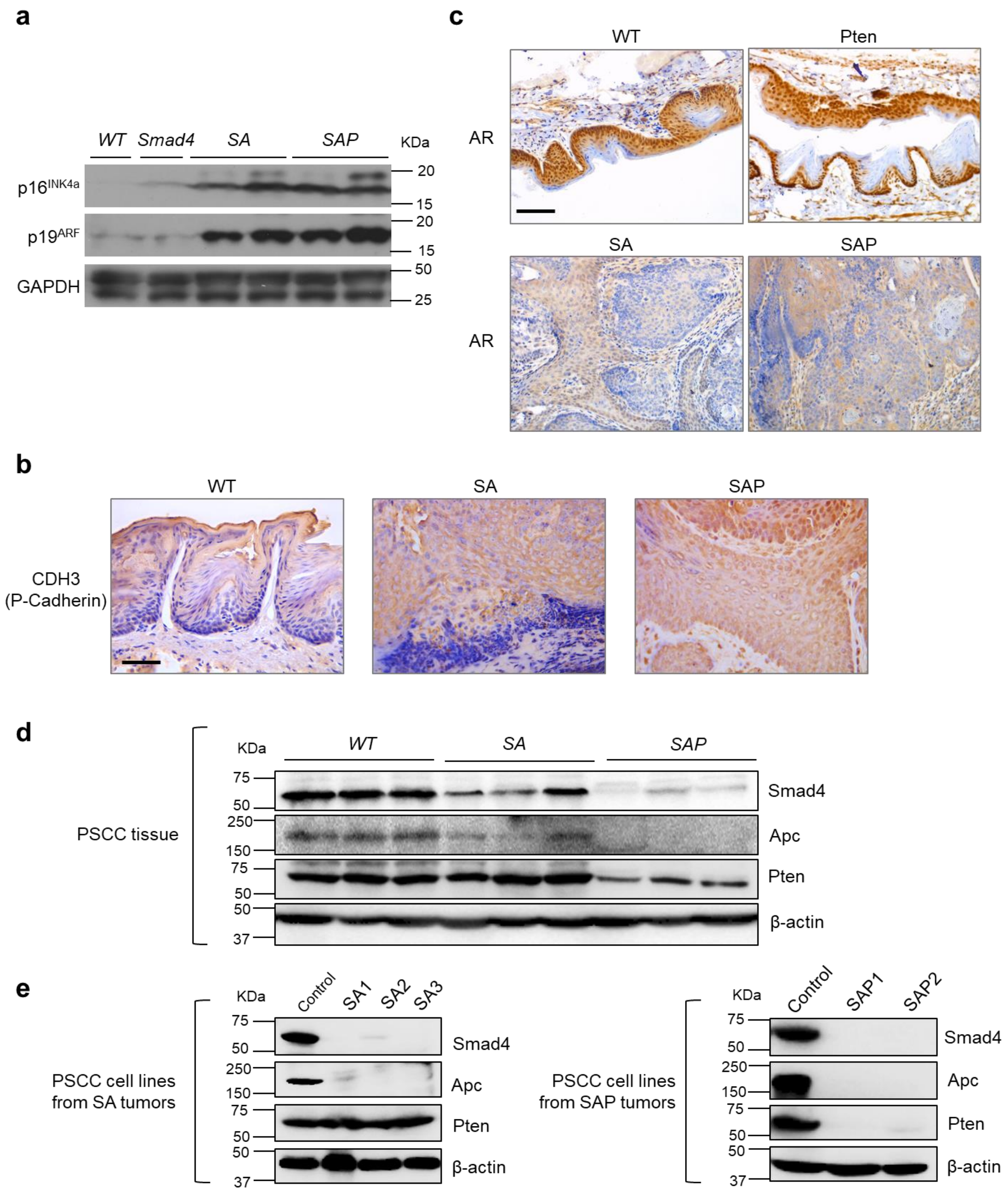

Supplementary Figure 6. Expression validation for upregulated and downregulated proteins in mouse PSCC. **(a)** Western blot showing the upregulation of $p16^{INK4a}$ and $p19^{ARF}$ in SA and SAP tumors compared with normal penises from WT or *PB-Cre4*$^{+}$ *Smad4*$^{L/L}$ mice (n = 3 for SA and SAP tumors). **(b)** Representative IHC images of CDH3 (P-Cadherin). Scale bar 50 μm. **(c)** Representative IHC images of AR. Scale bar 100 μm. **(d)** Expression level of Smad4, Apc and Pten in wild type (WT) penis, SA tumor and SAP tumors, detected by western blot (n = 3). **(e)** Expression level of Smad4, Apc and Pten in wild type penis tissue (control), 3 SA cell lines and 2 SAP cell lines, detected by western blot (n = 3).

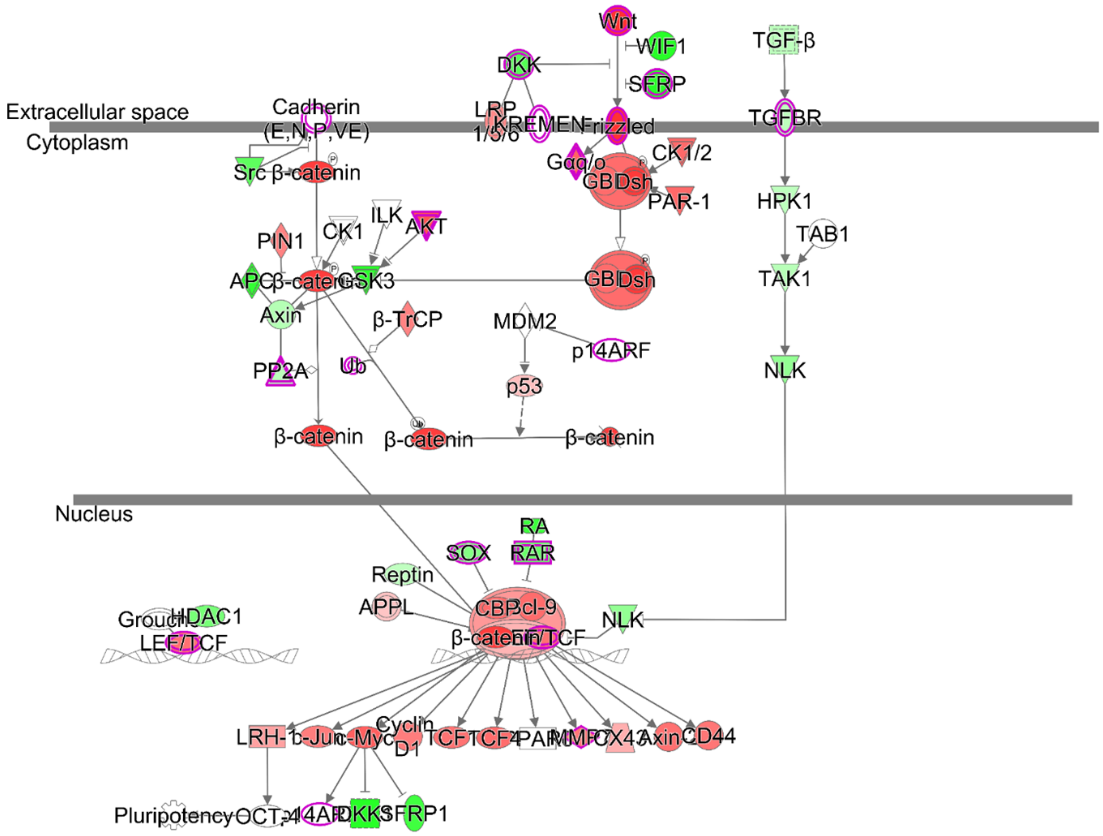

Supplementary Figure 7. Activated Wnt/β-catenin signaling in human penile cancer. Wnt/β-catenin signaling was identified as an activated pathway in human penile cancer compared with normal glans, based on IPA analysis of the transcriptome dataset GSE57955 (Kuasne et al., 2015). Red and green colors denote up- and down-regulated genes, respectively. Enrichment z-score = 1.3, *P* value = 0.00102.

SUPPLEMENTARY TABLES

Supplementary Table 1. Clinical and histological information of 8 archived PSCC samples at MD Anderson Cancer Center.

ID	Patient Age	Surgery Type	HPV status	Path Stage	Grade	Histology	cN stage
1000114	87	radical penectomy	negative	2	3	SCC basaloid	2
868816	68	radical penectomy	unknown	3	2	SCC, warty	0
770600	72	Partial penectomy	unknown	2	2	SCC	0
704741	45	Partial penectomy	unknown	1	3	SCC	1
630251	52	Partial penectomy	unknown	3	2	SCC	1
540479	31	Partial penectomy	unknown	2	3	SCC	0
417497	22	circumcision	unknown	unknown	unknown	unknown	3
405263	35	Partial penectomy	unknown	2	2	SCC	0

Supplementary Table 2. Differentially expressed genes between wild type penis and SA penile tumors. To reduce the size of the table, only genes with log2 (fold change) >6 or <-6 are shown. *P*-values obtained after multiple binomial tests were corrected and adjusted using BH method.

Gene	log2 (fold change)	P value	P_adjusted	Smad4/APC _mean	Control _mean	Regulation
4922501L14Rik	9.377880796	1.50E-30	3.90E-29	926.6074813	0.503109461	Up
Abca13	9.326697511	7.18E-132	5.07E-129	6372.669926	8.835299002	Up
Igfbpl1	9.31036525	4.59E-46	2.50E-44	1457.48232	1.526123863	Up
Ascl5	9.283830816	1.29E-22	2.21E-21	531.0784335	0	Up
Ces1f	9.232035225	5.23E-28	1.19E-26	892.7127959	0.503109461	Up
Mmp10	9.203370317	1.02E-74	1.37E-72	2399.211008	2.876626895	Up
Foxa2	9.133021984	7.76E-50	4.63E-48	1547.334433	1.754382112	Up
Hydin	9.111640493	5.00E-29	1.20E-27	736.3424582	0.511507201	Up
Cdh22	9.08453218	1.85E-21	2.93E-20	449.2416077	0	Up
Dkk4	8.88202079	3.82E-141	3.75E-138	6969.251532	13.52129871	Up
Cyp26c1	8.875553694	2.08E-26	4.35E-25	645.3451255	0.511507201	Up
Pla2g2a	8.850398497	1.91E-48	1.11E-46	1242.092332	1.883409978	Up
Bpifb4	8.796217542	2.78E-72	3.46E-70	3028.349123	5.546395287	Up
R3hdml	8.732986943	1.47E-26	3.11E-25	538.8400251	0.374081595	Up
Gp2	8.715954339	7.57E-34	2.40E-32	3601.512936	5.155518214	Up
Fgf15	8.694099125	3.47E-19	4.75E-18	329.1691573	0	Up
Abp1	8.654868816	1.47E-39	6.15E-38	888.274078	1.397095996	Up
Rab3c	8.562471819	1.16E-60	1.04E-58	1555.727155	3.288903714	Up
Cartpt	8.554479038	3.35E-106	1.26E-103	6124.21347	14.63135228	Up
Fgf20	8.513650875	2.44E-48	1.42E-46	1164.989681	2.38651944	Up
4921525O09Rik	8.39247137	3.55E-68	3.89E-66	1689.615713	4.157697031	Up
Grp	8.362063299	1.13E-79	1.80E-77	2516.293647	6.835863162	Up
Gm21002	8.356961679	2.53E-17	3.04E-16	255.0385604	0	Up
Akp3	8.353997837	1.98E-23	3.54E-22	413.1397502	0.374081595	Up
Krt73	8.349737143	1.79E-121	1.05E-118	16081.28135	44.72005067	Up
Mcpt1	8.310134081	9.75E-20	1.39E-18	1220.285268	1.380300517	Up
Padi3	8.305637691	2.01E-116	1.04E-113	5959.494391	17.12549984	Up
5730457N03Rik	8.24961797	1.06E-16	1.23E-15	237.3578787	0	Up
Krt71	8.234443544	2.50E-19	3.45E-18	1508.419417	1.883409978	Up
Fgf4	8.2187418	1.39E-88	2.91E-86	2502.015191	7.446600745	Up
Krt27	8.217958526	5.59E-35	1.89E-33	5184.515472	11.84555803	Up
Alpk2	8.199242537	1.24E-16	1.43E-15	217.9484731	0	Up
Shh	8.162218592	5.65E-133	4.34E-130	3272.551849	10.59807885	Up
Cbln1	8.154773496	3.50E-16	3.87E-15	220.8438323	0	Up
Npffr1	8.106552251	6.12E-28	1.40E-26	470.4173992	1.006218923	Up
Wnt7a	8.096679689	6.67E-46	3.59E-44	842.6690517	2.38651944	Up
Otop1	8.080331976	1.68E-87	3.42E-85	3215.03742	10.34002312	Up
Krt18	8.077951547	2.09E-157	2.63E-154	8129.103974	27.96402816	Up
Cpsf4l	8.061020922	1.46E-37	5.62E-36	710.1277397	1.762779851	Up
Krt26	8.06094597	2.25E-15	2.36E-14	223.6143894	0	Up
Odam	8.045388696	3.65E-51	2.25E-49	1210.986545	3.388134096	Up
Oprd1	8.010362016	1.87E-20	2.76E-19	598.1496286	1.023014402	Up
Scgb2b19	7.984427079	2.65E-15	2.77E-14	194.0499878	0	Up
Krt42	7.973866342	1.18E-199	4.16E-196	10071.17464	38.33845303	Up
Idi2	7.971764754	2.30E-104	8.27E-102	3762.353735	14.02901244	Up

Drd5	7.970508326	4.52E-15	4.65E-14	202.4916467	0	Up
Tlx2	7.957548146	4.78E-31	1.28E-29	548.9266921	1.380300517	Up
Igfn1	7.919802643	5.20E-33	1.55E-31	511.1348958	1.388698257	Up
Ctnna2	7.878680104	1.10E-26	2.34E-25	386.1329009	0.748163189	Up
Pkd2l1	7.855168207	8.68E-35	2.91E-33	616.2134556	1.771177591	Up
Cldn6	7.852196005	3.25E-25	6.49E-24	396.192847	0.877191056	Up
Cdx1	7.781172027	3.40E-26	7.04E-25	360.3548342	1.014616662	Up
Bpifb3	7.73370506	4.17E-14	4.03E-13	160.941044	0	Up
4930426D05Rik	7.726621799	1.02E-19	1.44E-18	254.637838	0.503109461	Up
Gpr55	7.715591179	4.63E-20	6.70E-19	244.7631336	0.503109461	Up
Pla2g10	7.715457415	6.15E-14	5.86E-13	164.5678951	0	Up
Cyp26a1	7.710974872	6.25E-19	8.40E-18	265.6280213	0.503109461	Up
Foxi3	7.691674797	1.19E-28	2.80E-27	452.3705066	1.388698257	Up
Cd200r2	7.677069505	3.29E-18	4.20E-17	270.057642	0.511507201	Up
Kng2	7.633473381	9.45E-14	8.88E-13	145.6059274	0	Up
Car3	7.598134701	1.51E-100	4.75E-98	2210.405769	10.20259751	Up
Krt72	7.586790829	1.74E-11	1.31E-10	317.0276712	0	Up
Mug1	7.56282616	1.69E-18	2.21E-17	226.6268632	0.374081595	Up
Gprc5d	7.553276308	2.11E-67	2.23E-65	2532.74967	11.58289803	Up
Pnoc	7.53145957	7.09E-24	1.29E-22	304.859417	1.014616662	Up
Cdkn2a	7.529790658	4.26E-55	3.04E-53	803.8434046	3.508764223	Up
Cd177	7.527243746	4.34E-17	5.14E-16	245.2517405	0.503109461	Up
Krt25	7.503323522	3.76E-22	6.24E-21	1912.383685	6.332753701	Up
Siglec15	7.500814708	3.04E-13	2.75E-12	130.1248559	0	Up
Adamts18	7.486815137	5.41E-23	9.42E-22	298.0630816	0.885588795	Up
Klhl14	7.475611092	1.78E-23	3.19E-22	287.7966534	0.748163189	Up
Padi4	7.463629327	1.26E-70	1.48E-68	1966.650924	9.416239099	Up
Chrnd	7.461507604	2.26E-18	2.91E-17	205.8510892	0.511507201	Up
Stac	7.456630257	1.44E-91	3.33E-89	2264.858913	11.42867694	Up
Serpinb3c	7.452880633	1.45E-51	9.13E-50	777.645008	3.921041042	Up
Mmp20	7.447069306	1.03E-17	1.27E-16	212.5918635	0.511507201	Up
Hrh3	7.42749358	1.15E-31	3.22E-30	445.9752573	1.754382112	Up
Cpb2	7.42558954	2.25E-16	2.54E-15	225.367748	0.374081595	Up
Crym	7.413510766	1.70E-18	2.23E-17	1710.39544	5.28454608	Up
Gbx2	7.398182299	7.33E-13	6.38E-12	118.0627118	0	Up
Strc	7.37769529	3.49E-51	2.15E-49	730.6911566	3.792013176	Up
Umod	7.377683305	1.03E-12	8.82E-12	118.3709368	0	Up
Myt1l	7.355545021	4.08E-27	8.92E-26	338.5029271	1.122244784	Up
Sstr5	7.355093931	1.30E-12	1.10E-11	116.3756148	0	Up
Csn1s1	7.343970925	3.12E-18	3.99E-17	1021.143428	3.288903714	Up
Mmp13	7.334883675	9.00E-72	1.09E-69	1575.395587	8.706271135	Up
Tchhl1	7.321048867	7.49E-63	7.22E-61	1369.628785	7.850479825	Up
Cacna1i	7.31068142	2.64E-39	1.09E-37	560.3790239	2.923219859	Up
Myh7b	7.301047586	2.65E-56	2.02E-54	1018.005554	5.692218633	Up
Evx1	7.28909787	8.79E-22	1.43E-20	258.6385155	1.014616662	Up
Sidt1	7.285988491	6.58E-36	2.32E-34	472.0012857	2.394917179	Up
Svopl	7.249683468	7.38E-250	1.30E-245	8424.76139	53.3170721	Up
Sp5	7.24708112	3.98E-113	1.90E-110	3131.954137	19.39138916	Up
Ubxn10	7.245626897	2.62E-17	3.14E-16	172.2519876	0.511507201	Up
Krt28	7.234841707	1.73E-30	4.48E-29	1596.019683	7.829080079	Up

Dner	7.232328009	4.26E-84	7.67E-82	1489.727122	8.572639002	Up
Kif12	7.222572435	3.43E-12	2.77E-11	99.89304325	0	Up
U90926	7.215147198	1.33E-32	3.89E-31	445.1401847	1.870407973	Up
Mug-ps1	7.205858686	6.38E-12	5.04E-11	106.3063746	0	Up
Cck	7.193209417	1.47E-26	3.10E-25	486.7931129	2.265889313	Up
Spo11	7.188988566	2.21E-86	4.39E-84	1381.558007	8.577243268	Up
Irx4	7.172594379	7.93E-62	7.32E-60	2141.332567	12.84256842	Up
Dnahc17	7.14025944	2.50E-108	9.59E-106	2089.564082	14.25727069	Up
Mcpt2	7.134660793	1.28E-14	1.27E-13	803.0402127	2.549138265	Up
Krt8	7.130503172	3.04E-135	2.43E-132	8398.503451	56.47694795	Up
Cntn3	7.125746126	8.95E-12	6.98E-11	92.87646591	0	Up
Sox2	7.099494762	5.74E-94	1.51E-91	2927.168083	19.84944815	Up
Ibsp	7.085229571	2.78E-18	3.57E-17	348.7324499	1.388698257	Up
Col25a1	7.079595293	2.63E-26	5.48E-25	359.7455361	1.900205458	Up
Bcl2l14	7.079005026	1.74E-37	6.60E-36	471.4165056	2.889628901	Up
Sorcs3	7.058456023	2.86E-31	7.81E-30	414.5856187	2.540740525	Up
Upk3a	7.051442052	1.70E-11	1.29E-10	88.27487681	0	Up
Nkx1-2	7.043258117	2.32E-11	1.74E-10	91.11105998	0	Up
Il24	7.022815634	2.25E-11	1.69E-10	85.49001751	0	Up
Fgf3	6.970253889	1.24E-35	4.29E-34	498.4854371	2.876626895	Up
Apoc4	6.964972496	4.52E-15	4.65E-14	145.9467722	0.374081595	Up
Tph2	6.956889668	4.17E-11	3.06E-10	82.7163367	0	Up
Amtn	6.937939207	3.71E-23	6.51E-22	333.588154	1.762779851	Up
Ascl1	6.937234546	2.56E-22	4.28E-21	263.0510548	1.397095996	Up
Prss56	6.933702216	2.81E-23	4.97E-22	258.2098005	1.509328384	Up
Sftpd	6.933407991	1.74E-15	1.85E-14	137.7278879	0.511507201	Up
Zfp804a	6.932519557	6.53E-11	4.71E-10	82.89561874	0	Up
Entpd8	6.928947798	1.24E-14	1.24E-13	144.7616839	0.374081595	Up
Snhg11	6.913533263	9.88E-197	2.91E-193	4170.334223	33.17372628	Up
Defa22	6.90841913	8.58E-11	6.14E-10	83.55637785	0	Up
Slc44a5	6.902975628	5.17E-31	1.39E-29	360.6295357	2.411712659	Up
Defa21	6.899171819	2.45E-14	2.40E-13	147.3426075	0.503109461	Up
Pnliprp2	6.886639578	4.26E-12	3.41E-11	343.4276393	1.006218923	Up
Ddx25	6.844848044	1.14E-10	8.08E-10	75.89680007	0	Up
Krt79	6.815708241	2.52E-43	1.23E-41	11587.72061	87.3726728	Up
9530053A07Rik	6.804431913	1.55E-10	1.08E-09	73.00880651	0	Up
Vil1	6.791264501	2.23E-32	6.49E-31	384.8405596	2.639970907	Up
Wdr72	6.791014091	1.68E-10	1.17E-09	71.20297913	0	Up
Gm6792	6.786343244	1.64E-10	1.15E-09	70.96059712	0	Up
Kcnk16	6.758155085	1.06E-14	1.07E-13	118.9992636	0.503109461	Up
Grm8	6.75594417	2.53E-10	1.73E-09	71.15740683	0	Up
Bex1	6.750544952	5.60E-27	1.22E-25	268.3932666	1.891807718	Up
Gm4792	6.742941681	1.47E-14	1.46E-13	117.8106123	0.511507201	Up
Stfa2	6.740627004	3.80E-52	2.43E-50	670.1542725	5.692218633	Up
Naip1	6.738911524	1.59E-27	3.54E-26	321.6475644	1.999435839	Up
Lef1	6.73475151	1.03E-103	3.62E-101	2084.693442	18.79744705	Up
Defa20	6.724800692	3.01E-10	2.04E-09	68.6963512	0	Up
Ptch2	6.714409402	1.22E-174	2.38E-171	4726.695839	43.28549115	Up
2810459M11Rik	6.690123389	4.06E-10	2.72E-09	66.95370029	0	Up
Gm12603	6.665624091	4.45E-10	2.97E-09	64.24441716	0	Up

Zp2	6.652990926	5.04E-10	3.34E-09	63.77103494	0	Up
Ppapdc1a	6.644756959	6.37E-10	4.18E-09	65.9012636	0	Up
Akr1d1	6.64228467	2.32E-13	2.11E-12	118.630051	0.511507201	Up
Bpifb5	6.641309578	1.21E-20	1.81E-19	206.6084095	1.25127265	Up
Avil	6.633686719	1.79E-88	3.72E-86	3090.311417	28.97864482	Up
Galnt13	6.612326448	5.22E-13	4.62E-12	116.890124	0.511507201	Up
Crhr1	6.586842991	2.66E-17	3.19E-16	150.3407414	0.748163189	Up
Hao1	6.578289756	1.84E-20	2.72E-19	199.1580743	1.526123863	Up
Itln1	6.575949892	1.07E-09	6.85E-09	61.06693891	0	Up
Gm11128	6.572746165	2.87E-09	1.75E-08	74.62539479	0	Up
Gpr179	6.567117283	1.94E-26	4.07E-25	288.5542148	2.403314919	Up
Nr2e3	6.56353228	1.31E-09	8.28E-09	61.50389154	0	Up
Cntn4	6.562103698	3.43E-13	3.09E-12	105.7219943	0.374081595	Up
D630010B17Rik	6.53441501	1.39E-09	8.74E-09	58.64883246	0	Up
Il1b	6.531749579	1.17E-29	2.93E-28	2158.319676	18.4583966	Up
Slc22a29	6.513703343	1.60E-09	1.00E-08	57.12563666	0	Up
Eps8l3	6.511246468	1.87E-16	2.13E-15	148.5472546	1.014616662	Up
Chi3l1	6.494293852	1.87E-51	1.17E-49	1147.117353	11.21721418	Up
Ap3b2	6.489783375	1.63E-17	1.99E-16	196.4452992	1.25127265	Up
Klk6	6.487603078	4.79E-90	1.07E-87	2018.893908	21.35037879	Up
Pnliprp1	6.468300013	2.99E-11	2.22E-10	221.6294697	1.014616662	Up
Lhx2	6.460949248	7.14E-47	3.96E-45	503.7285608	5.214302391	Up
Aadacl3	6.449262554	2.93E-09	1.79E-08	56.45348861	0	Up
Il19	6.446951926	2.63E-22	4.39E-21	221.9923396	1.754382112	Up
Hpd	6.437775246	1.39E-12	1.18E-11	100.0028099	0.511507201	Up
Elfn1	6.43170134	1.43E-28	3.35E-27	297.5592059	2.777396513	Up
Krt82	6.427461896	3.85E-12	3.09E-11	104.1148074	0.511507201	Up
Fgf5	6.425858648	1.00E-12	8.63E-12	96.74808088	0.503109461	Up
H2-T3	6.409334165	5.80E-09	3.44E-08	58.92070583	0	Up
Cxcl2	6.39427119	5.91E-24	1.09E-22	1063.479977	9.687296839	Up
Gpha2	6.39152182	1.43E-38	5.71E-37	404.1631167	4.16609477	Up
Gng3	6.377420276	4.39E-33	1.32E-31	369.5031115	3.783615436	Up
Akr1c18	6.367235073	5.63E-24	1.04E-22	301.2649061	2.785794253	Up
Tchh	6.36048596	6.90E-129	4.51E-126	58389.06987	682.1084906	Up
Jakmip2	6.347126129	1.35E-50	8.20E-49	662.1449777	7.196942751	Up
Reg3g	6.345414818	4.49E-23	7.85E-22	200.7394225	1.891807718	Up
Mpped1	6.331859947	9.17E-17	1.06E-15	509.5143522	3.869843812	Up
Slc45a1	6.327601092	1.53E-12	1.29E-11	87.25617721	0.511507201	Up
Trem1	6.321290093	1.51E-25	3.04E-24	321.2373608	3.014052502	Up
Spp1	6.315790887	6.20E-140	5.47E-137	11656.64404	141.2959805	Up
Abo	6.308709471	7.64E-09	4.46E-08	49.23884555	0	Up
Reg3d	6.291748853	9.29E-09	5.38E-08	48.6124574	0	Up
Cml3	6.26708697	4.24E-48	2.44E-46	1076.62637	12.19363561	Up
Abcg8	6.252704379	1.26E-08	7.22E-08	48.3296642	0	Up
Gad1	6.251539263	6.12E-53	4.02E-51	541.5037257	6.698437556	Up
Mmp9	6.249908404	2.37E-114	1.16E-111	5490.033328	69.08367118	Up
Lrrtm1	6.226451041	9.48E-27	2.03E-25	346.5928733	3.792013176	Up
Col22a1	6.223431389	4.61E-24	8.53E-23	223.2384299	2.523945046	Up
Pycr1	6.215793872	1.79E-102	6.08E-100	3647.972424	46.21475547	Up
B4galnt2	6.209331847	4.87E-74	6.33E-72	1149.844716	14.38548776	Up

Prdm6	6.17813881	1.44E-34	4.75E-33	492.5232035	5.524995542	Up
Hecw1	6.175134705	1.11E-11	8.55E-11	78.85439581	0.503109461	Up
Syt14	6.150023565	2.66E-08	1.47E-07	44.06078168	0	Up
Cadps	6.140482877	1.86E-92	4.56E-90	2149.131015	28.73738457	Up
Mapk4	6.112321113	9.40E-63	9.01E-61	896.3359559	12.31201476	Up
Gstm7	6.110807132	2.15E-138	1.81E-135	2574.142544	36.1717941	Up
Ang2	6.1048253	3.69E-08	2.00E-07	43.26745919	0	Up
Gm5483	6.093401854	4.49E-08	2.41E-07	42.73932572	0	Up
Muc6	6.09225702	1.28E-42	6.05E-41	481.0551771	5.778447009	Up
Tspan1	6.090303619	5.78E-10	3.81E-09	312.249076	2.012437845	Up
Clec4e	6.089405503	5.34E-22	8.77E-21	276.805195	3.014052502	Up
Ceacam2	6.074712648	8.19E-11	5.86E-10	74.65223538	0.374081595	Up
Fpr1	6.071549461	5.86E-08	3.10E-07	43.21142593	0	Up
Igf2bp1	6.063232285	3.19E-11	2.36E-10	70.88673895	0.374081595	Up
Col2a1	6.050082814	3.22E-24	6.01E-23	193.4103225	2.540740525	Up
Rhox5	6.045415259	1.45E-10	1.02E-09	76.47483667	0.511507201	Up
Htr5a	6.040531023	7.32E-08	3.83E-07	43.07181157	0	Up
Dpysl4	6.038073357	2.88E-111	1.21E-108	2049.935573	29.85583588	Up
Hsd17b2	6.016395097	3.35E-19	4.59E-18	160.7686513	1.633751985	Up
Defa5	6.011881021	6.43E-08	3.39E-07	38.96355759	0	Up
D130043K22Rik	6.004619302	6.81E-14	6.47E-13	101.1390689	1.014616662	Up
Krtap21-1	-6.044695991	2.46E-21	3.86E-20	213.4852023	18128.56341	Down
Dlx6	-6.11748125	4.45E-56	3.34E-54	3.528327514	300.6893804	Down
Tmprss11c	-6.397464533	2.25E-87	4.52E-85	6.176664673	496.9579304	Down
Slco1a4	-6.425504338	2.33E-12	1.92E-11	0.371486554	44.87265017	Down
Spink6	-6.478763741	2.13E-167	3.42E-164	76.38478648	6852.275358	Down
Cytl1	-6.482651424	1.10E-33	3.44E-32	1.281949897	159.3436428	Down
Sult1e1	-6.61534326	1.43E-18	1.88E-17	10.65522572	1665.79523	Down
Lypd8	-6.714414615	7.62E-27	1.64E-25	26.90020804	3804.004581	Down
Gm10228	-7.020986015	1.44E-22	2.46E-21	37.33891952	7101.542159	Down
Krtap6-1	-7.139023196	6.71E-23	1.16E-21	29.59197377	6190.506016	Down
Ffar3	-7.182132771	9.49E-12	7.38E-11	0	44.41694447	Down
Gm10229	-7.412088728	2.84E-28	6.56E-27	45.95409694	11056.80266	Down
Krtap22-2	-7.498046197	2.71E-27	5.96E-26	16.71594643	4357.667886	Down
Tdo2	-7.549333643	3.28E-51	2.03E-49	1.227883018	402.1842283	Down
Krtap6-5	-7.644237122	1.74E-37	6.60E-36	33.52765292	8726.079639	Down
1110025L11Rik	-7.963260973	1.87E-29	4.62E-28	13.79208778	5191.986411	Down
Myoc	-9.147676473	3.06E-119	1.69E-116	2.102583775	1675.245304	Down

Supplementary Table 3. Enriched canonical pathways in SA tumors relative to normal penis by IPA. *P* values by Right-Tailed Fisher's Exact Test.

Ingenuity Canonical Pathways	P value	Ratio	z-score	Downregulated	Upregulated
Granulocyte Adhesion and Diapedesis	5.01187E-12	0.266	#NUM!	9/177 (5%)	38/177 (21%)
Agranulocyte Adhesion and Diapedesis	6.30957E-11	0.249	#NUM!	14/189 (7%)	33/189 (17%)
Inhibition of Matrix Metalloproteases	0.00000001	0.436	#NUM!	2/39 (5%)	15/39 (38%)
Role of Osteoblasts, Osteoclasts and Chondrocytes in Rheumatoid Arthritis	1.09648E-08	0.215	#NUM!	9/219 (4%)	38/219 (17%)
Hepatic Fibrosis / Hepatic Stellate Cell Activation	2.63027E-08	0.224	#NUM!	10/183 (5%)	31/183 (17%)
Atherosclerosis Signaling	9.54993E-08	0.25	#NUM!	6/124 (5%)	25/124 (20%)
Bladder Cancer Signaling	3.71535E-07	0.276	#NUM!	5/87 (6%)	19/87 (22%)
Cellular Effects of Sildenafil (Viagra)	2.5704E-06	0.225	#NUM!	11/129 (9%)	18/129 (14%)
Basal Cell Carcinoma Signaling	3.01995E-06	0.278	3.153	2/72 (3%)	18/72 (25%)
LPS/IL-1 Mediated Inhibition of RXR Function	5.01187E-06	0.186	0.535	11/221 (5%)	30/221 (14%)
Eicosanoid Signaling	7.76247E-06	0.281	1	5/64 (8%)	13/64 (20%)
cAMP-mediated signaling	9.77237E-06	0.183	3.042	6/219 (3%)	34/219 (16%)
Amyotrophic Lateral Sclerosis Signaling	1.28825E-05	0.235	#NUM!	4/98 (4%)	19/98 (19%)
Serotonin Receptor Signaling	1.69824E-05	0.318	#NUM!	3/44 (7%)	11/44 (25%)
Role of Macrophages, Fibroblasts and Endothelial Cells in Rheumatoid Arthritis	3.54813E-05	0.162	#NUM!	9/296 (3%)	39/296 (13%)
Human Embryonic Stem Cell Pluripotency	4.57088E-05	0.201	#NUM!	7/134 (5%)	20/134 (15%)
LXR/RXR Activation	5.62341E-05	0.207	-2.132	5/121 (4%)	20/121 (17%)
Colorectal Cancer Metastasis Signaling	5.7544E-05	0.169	4.11	6/236 (3%)	34/236 (14%)
Differential Regulation of Cytokine Production in Macrophages and T Helper Cells by IL-17A and IL-17F	7.58578E-05	0.444	#NUM!	0/18 (0%)	8/18 (44%)
Axonal Guidance Signaling	7.94328E-05	0.145	#NUM!	12/434 (3%)	51/434 (12%)
Glutamate Receptor Signaling	0.000102329	0.263	2.121	2/57 (4%)	13/57 (23%)
Gαi Signaling	0.000131826	0.2	1.528	3/120 (3%)	21/120 (18%)
G-Protein Coupled Receptor Signaling	0.000169824	0.16	#NUM!	7/256 (3%)	34/256 (13%)
GABA Receptor Signaling	0.00020893	0.239	-0.277	4/67 (6%)	12/67 (18%)
Airway Pathology in Chronic Obstructive Pulmonary Disease	0.000251189	0.625	#NUM!	0/8 (0%)	5/8 (63%)
FXR/RXR Activation	0.000288403	0.19	#NUM!	6/126 (5%)	18/126 (14%)
eNOS Signaling	0.000316228	0.183	1.342	11/142 (8%)	15/142 (11%)
Dopamine-DARPP32 Feedback in cAMP Signaling	0.000457088	0.174	0.784	10/161 (6%)	18/161 (11%)
Gαs Signaling	0.00057544	0.193	2.324	4/109 (4%)	17/109 (16%)
Coagulation System	0.000707946	0.286	0	1/35 (3%)	9/35 (26%)
GPCR-Mediated Nutrient Sensing in Enteroendocrine Cells	0.001047129	0.202	#NUM!	5/84 (6%)	12/84 (14%)

Gap Junction Signaling	0.001230269	0.168	#NUM!	11/155 (7%)	15/155 (10%)
GPCR-Mediated Integration of Enteroendocrine Signaling Exemplified by an L Cell	0.00128825	0.211	#NUM!	3/71 (4%)	12/71 (17%)
Synaptic Long Term Depression	0.001698244	0.169	1.633	8/142 (6%)	16/142 (11%)
Role of NANOG in Mammalian Embryonic Stem Cell Pluripotency	0.001819701	0.18	1.342	5/111 (5%)	15/111 (14%)
Hepatic Cholestasis	0.002137962	0.161	#NUM!	3/161 (2%)	23/161 (14%)
Sperm Motility	0.002290868	0.174	0.229	8/121 (7%)	13/121 (11%)
TREM1 Signaling	0.002290868	0.2	3.873	0/75 (0%)	15/75 (20%)
Endothelin-1 Signaling	0.002691535	0.157	0.426	9/172 (5%)	18/172 (10%)
Leukocyte Extravasation Signaling	0.002818383	0.152	1.877	9/198 (5%)	21/198 (11%)
Actin Cytoskeleton Signaling	0.003019952	0.148	1.886	9/216 (4%)	23/216 (11%)
Differential Regulation of Cytokine Production in Intestinal Epithelial Cells by IL-17A and IL-17F	0.003019952	0.304	#NUM!	0/23 (0%)	7/23 (30%)
Antiproliferative Role of Somatostatin Receptor 2	0.003311311	0.206	0.447	4/63 (6%)	9/63 (14%)
HIF1α Signaling	0.003801894	0.176	#NUM!	4/102 (4%)	14/102 (14%)
Role of IL-17A in Psoriasis	0.003890451	0.385	#NUM!	0/13 (0%)	5/13 (38%)
Corticotropin Releasing Hormone Signaling	0.004168694	0.171	1.414	4/111 (4%)	15/111 (14%)
Wnt/β-catenin Signaling	0.004265795	0.154	0.816	5/169 (3%)	21/169 (12%)
Gustation Pathway	0.005011872	0.165	#NUM!	4/121 (3%)	16/121 (13%)
Prostanoid Biosynthesis	0.005495409	0.444	#NUM!	2/9 (22%)	2/9 (22%)
Ovarian Cancer Signaling	0.005888437	0.16	#NUM!	5/131 (4%)	16/131 (12%)
Protein Citrullination	0.00616595	0.6	#NUM!	0/5 (0%)	3/5 (60%)
Dermatan Sulfate Biosynthesis (Late Stages)	0.006456542	0.217	#NUM!	3/46 (7%)	7/46 (15%)
Nitric Oxide Signaling in the Cardiovascular System	0.007079458	0.17	1.213	7/100 (7%)	10/100 (10%)
Cardiac β-adrenergic Signaling	0.007079458	0.158	3	4/133 (3%)	17/133 (13%)
Retinoate Biosynthesis I	0.00724436	0.242	#NUM!	2/33 (6%)	6/33 (18%)
Clathrin-mediated Endocytosis Signaling	0.007413102	0.146	#NUM!	9/185 (5%)	18/185 (10%)
Glutamate Dependent Acid Resistance	0.007943282	1	#NUM!	0/2 (0%)	2/2 (100%)
Sertoli Cell-Sertoli Cell Junction Signaling	0.008317638	0.146	#NUM!	10/178 (6%)	16/178 (9%)
Relaxin Signaling	0.008317638	0.156	1.291	5/135 (4%)	16/135 (12%)
VDR/RXR Activation	0.00851138	0.179	1.89	2/78 (3%)	12/78 (15%)
Retinol Biosynthesis	0.008709636	0.235	#NUM!	1/34 (3%)	7/34 (21%)
Chondroitin Sulfate Biosynthesis (Late Stages)	0.008709636	0.208	#NUM!	3/48 (6%)	7/48 (15%)
Extrinsic Prothrombin Activation Pathway	0.010715193	0.312	1.342	1/16 (6%)	4/16 (25%)
Serotonin and Melatonin Biosynthesis	0.011481536	0.5	#NUM!	0/6 (0%)	3/6 (50%)
Phospholipases	0.012589254	0.19	#NUM!	4/58 (7%)	7/58 (12%)
Regulation of the Epithelial-Mesenchymal Transition Pathway	0.012589254	0.141	#NUM!	6/184 (3%)	20/184 (11%)

Role of Pattern Recognition Receptors in Recognition of Bacteria and Viruses	0.014791084	0.152	2.53	1/125 (1%)	18/125 (14%)
Heparan Sulfate Biosynthesis (Late Stages)	0.015135612	0.192	#NUM!	3/52 (6%)	7/52 (13%)
Neuropathic Pain Signaling In Dorsal Horn Neurons	0.015488166	0.16	2	4/100 (4%)	12/100 (12%)
Estrogen Biosynthesis	0.016982437	0.211	#NUM!	5/38 (13%)	3/38 (8%)
CREB Signaling in Neurons	0.017378008	0.14	3.13	3/171 (2%)	21/171 (12%)
Role of Oct4 in Mammalian Embryonic Stem Cell Pluripotency	0.018620871	0.196	1	1/46 (2%)	8/46 (17%)
Bupropion Degradation	0.019952623	0.24	#NUM!	5/25 (20%)	1/25 (4%)
nNOS Signaling in Neurons	0.021379621	0.191	0.447	3/47 (6%)	6/47 (13%)
T Helper Cell Differentiation	0.022387211	0.169	#NUM!	1/71 (1%)	11/71 (15%)
Thyronamine and Iodothyronamine Metabolism	0.022387211	0.667	#NUM!	0/3 (0%)	2/3 (67%)
Thyroid Hormone Metabolism I (via Deiodination)	0.022387211	0.667	#NUM!	0/3 (0%)	2/3 (67%)
PCP pathway	0.022387211	0.175	1.897	3/63 (5%)	8/63 (13%)
Neuroprotective Role of THOP1 in Alzheimer's Disease	0.022908677	0.2	#NUM!	4/40 (10%)	4/40 (10%)
Chondroitin Sulfate Biosynthesis	0.025118864	0.179	#NUM!	3/56 (5%)	7/56 (13%)
Protein Kinase A Signaling	0.02630268	0.119	-0.686	10/386 (3%)	36/386 (9%)
Calcium Signaling	0.026915348	0.135	1.155	10/178 (6%)	14/178 (8%)
Acute Phase Response Signaling	0.026915348	0.136	2.673	6/169 (4%)	17/169 (10%)
Acetone Degradation I (to Methylglyoxal)	0.028840315	0.222	#NUM!	5/27 (19%)	1/27 (4%)
Melanocyte Development and Pigmentation Signaling	0.034673685	0.155	1.897	4/84 (5%)	9/84 (11%)
Heparan Sulfate Biosynthesis	0.034673685	0.169	#NUM!	3/59 (5%)	7/59 (12%)
Dermatan Sulfate Biosynthesis	0.034673685	0.169	#NUM!	3/59 (5%)	7/59 (12%)
Synaptic Long Term Potentiation	0.034673685	0.143	2.668	6/119 (5%)	11/119 (9%)
FGF Signaling	0.03801894	0.153	1.387	4/85 (5%)	9/85 (11%)
Role of IL-17F in Allergic Inflammatory Airway Diseases	0.038904514	0.182	2.646	0/44 (0%)	8/44 (18%)
The Visual Cycle	0.038904514	0.267	#NUM!	1/15 (7%)	3/15 (20%)
Intrinsic Prothrombin Activation Pathway	0.039810717	0.207	2.236	0/29 (0%)	6/29 (21%)
Glutathione-mediated Detoxification	0.039810717	0.207	#NUM!	1/29 (3%)	5/29 (17%)
Uracil Degradation II (Reductive)	0.042657952	0.5	#NUM!	1/4 (25%)	1/4 (25%)
Retinoate Biosynthesis II	0.042657952	0.5	#NUM!	0/4 (0%)	2/4 (50%)
α-tocopherol Degradation	0.042657952	0.5	#NUM!	0/4 (0%)	2/4 (50%)
Thymine Degradation	0.042657952	0.5	#NUM!	1/4 (25%)	1/4 (25%)
Phototransduction Pathway	0.043651583	0.17	#NUM!	3/53 (6%)	6/53 (11%)
α-Adrenergic Signaling	0.044668359	0.149	1	3/87 (3%)	10/87 (11%)
Superpathway of Melatonin Degradation	0.046773514	0.161	#NUM!	8/62 (13%)	2/62 (3%)
Altered T Cell and B Cell Signaling in Rheumatoid Arthritis	0.047863009	0.148	#NUM!	1/88 (1%)	12/88 (14%)
Role of Cytokines in Mediating Communication between Immune Cells	0.047863009	0.167	#NUM!	0/54 (0%)	9/54 (17%)

Supplementary Table 4. Examples of significantly enriched pathways with genes in SA tumors compared with normal penis. *P*-values obtained after multiple binomial tests were corrected and adjusted using BH method.

Gene Symbol	Fold Change	Adjusted P value		Gene Symbol	Fold Change	Adjusted P value
Granulocyte/Agranulocyte Adhesion and Diapedesis (P value = 5.0E-12/6.3E-11)				*Matrix Metalloproteases (P value = 1.0E-08)*		
Il1b	92.52	2.93E-28		Mmp10	589.51	1.37E-72
Cxcl2	84.11	1.09E-22		Mmp20	174.50	1.27E-16
Csf3	30.50	2.28E-12		Mmp13	161.44	1.09E-69
Cxcl3	30.40	8.20E-16		Mmp9	76.10	1.16E-111
Ccl4	28.81	6.35E-12		Mmp3	59.28	3.67E-117
Ccl3	27.04	4.00E-16		Mmp7	49.78	3.20E-40
Cxcr4	24.91	2.40E-78		Mmp1a	46.87	4.10E-08
Cxcl5	12.90	1.60E-26		Mmp12	30.93	3.46E-32
Tnf	10.06	1.00E-14		Mmp8	17.95	7.22E-06
Cxcl1	5.34	2.08E-15		Adam12	8.52	4.39E-33
Ccl2	4.96	6.47E-11		Mmp25	6.30	3.10E-06
Eicosanoid Signaling (P value = 7.8E-06)				*Wnt/β-catenin Signaling (P value = 4.3E-03)*		
Pla2g2a	461.57	1.11E-46		Dkk4	471.80	3.75E-138
Pla2g10	210.18	5.86E-13		Wnt7a	273.74	3.59E-44
Pla2g4e	47.28	1.49E-149		Sox2	137.14	1.51E-91
Pla2g2c	25.90	0.000343191		Lef1	106.50	3.62E-101
Alox15	14.10	7.97E-08		Wnt10b	32.60	1.29E-54
Alox8	10.15	7.12E-17		Dkk1	20.88	9.50E-05
Ptgs2	9.31	1.41E-05		Wnt16	20.30	1.06E-65
Pla2g7	7.46	1.78E-51		Wnt6	17.58	2.59E-92
Hpgds	5.25	6.50E-32		Axin2	14.15	3.89E-90
Pla2g2d	0.22	6.69E-09		Wnt9b	9.26	3.58E-10
Plb1	0.16	1.12E-32		Tcf7	9.23	1.09E-48
Pla2g3	0.13	8.68E-94		Wnt5a	5.26	5.02E-36
Ptgis	0.12	3.63E-21		Wnt3	0.25	3.08E-20
Ptgds	0.09	6.28E-12		Wnt2	0.12	1.28E-11
FGF Signaling (P value = 3.8E-02)						
Fgf15	414.18	4.75E-18				
Fgf20	365.48	1.42E-46				
Fgf4	297.91	2.91E-86				
Fgf3	125.39	4.29E-34				
Fgf5	85.98	8.63E-12				
Fgf8	39.06	8.45E-10				
Fgfrl1	11.71	6.92E-64				
Fgf9	4.99	7.18E-18				
Fgf10	0.22	5.38E-15				
Fgfr3	0.19	1.83E-93				

Supplementary Table 5. Regulator effects identified by IPA by comparing SA tumors compared with normal penis. Top 50 lines are shown.

ID	Consistency Score	Regulator	Target Total	Target Molecules in Dataset	Diseases & Functions
1	4.25	PTGS2	16	Ccl2,CCL3L3,CCL4,CCR7,CXCL3,CXCR4,FN1,IL11,IL1B,IL6,MSR1,PIK3CG,SELL,TGM2,TNF,TNFSF11	adhesion of blood cells
2	4.25	PTGS2	16	Ccl2,CCL3L3,CCL4,CCR7,CXCL3,CXCR4,FN1,IL11,IL1B,IL6,MSR1,PIK3CG,SELL,TGM2,TNF,TNFSF11	adhesion of immune cells
3	4.007	PTGS2	18	Ccl2,CCL4,CCR7,CHGA,CSF3,CXCL3,FN1,IL1B,IL23A,IL6,MMP1,MMP9,MSR1,PIK3CG,SELL,TGM2,TNF,TNFSF11	activation of blood cells
4	4	peptidoglycan	16	BCL2A1,Ccl2,CCL3L3,CSF3,CXCL2,FPR2,IL12B,IL17A,IL1B,IL23A,IL6,MMP1,PTGS2,S100A8,S100A9,TNF	generation of cells
5	3.881	E. coli B4 lipopolysaccharide	17	C5,Ccl2,CCL3L3,CCL4,CSF3,CXCL2,CXCL3,CXCL6,FPR1,FPR2,IL12B,IL17A,IL1B,IL6,PLAUR,PTGS2,TNF	migration of phagocytes
6	3.881	PTGS2	17	Ccl2,CCL4,CCR7,CHGA,CSF3,CXCL3,FN1,IL1B,IL23A,IL6,MMP9,MSR1,PIK3CG,SELL,TGM2,TNF,TNFSF11	activation of leukocytes
7	3.878	poly rI:rC-RNA	24	Ccl2,CCL3L3,CCL4,CCR7,CEACAM1,CSF3,CXCL2,CXCL3,CXCR4,FN1,FPR1,IL12B,IL17A,IL1B,IL6,ITGAX,MMP9,NLRP3,PIGR,PLAUR,PRKCQ,PTGS2,TNF,WNT5A	migration of phagocytes
8	3.873	E. coli B4 lipopolysaccharide	15	C5,Ccl2,CCL3L3,CCL4,CXCL2,CXCL3,CXCL6,FPR1,FPR2,IL12B,IL17A,IL1B,IL23A,PLAUR,TNF	chemotaxis of myeloid cells
9	3.838	PI3K (complex)	22	Ccl2,CCL4,CCR1,CXCR4,FN1,GLI1,IL12B,IL17A,IL17F,IL1B,IL23A,IL6,MMP1,MMP12,MMP9,PIK3CG,PTGS2,SELL,SPP1,TGM2,TNF,TREM1	cell movement of blood cells
10	3.838	PI3K (complex)	22	Ccl2,CCL4,CCR1,CXCR4,FN1,GLI1,IL12B,IL17A,IL17F,IL1B,IL23A,IL6,MMP1,MMP12,MMP9,PIK3CG,PTGS2,SELL,SPP1,TGM2,TNF,TREM1	leukocyte migration
11	3.771	MAPK14	18	Ccl2,CCR7,CDKN2A,FN1,IL1B,IL6,KITLG,MMP1,MMP13,MMP3,MMP9,NPY,PLAUR,PTGS2,TGM2,TIMP1,TNF,TNN	vasculogenesis
12	3.771	trinitrobenzenesulfonic acid	18	Ccl2,CCL4,CLEC4E,CSF3,CXCL3,CXCL6,IL12B,IL17A,IL1B,IL23A,IL6,MMP9,PTGS2,S100A8,S100A9,Saa3,TACR1,TNF	activation of leukocytes
13	3.75	PTGS2	16	Ccl2,CCL4,CCR7,CHGA,CSF3,CXCL3,FN1,IL1B,IL23A,IL6,MMP9,MSR1,SELL,TGM2,TNF,TNFSF11	activation of phagocytes
14	3.75	PTGS2	16	Ccl2,CCL3L3,CCL4,CCR7,CSF3,CXCL3,CXCR4,FN1,IL1B,IL6,MMP9,PIK3CG,SELL,TGM2,TNF,TNFSF11	cell movement of mononuclear leukocytes
15	3.671	PI3K (complex)	19	Ccl2,CCL4,CCR1,CXCR4,FN1,IL12B,IL17A,IL1B,IL23A,IL6,MMP12,MMP9,PIK3CG,PTGS2,SELL,SPP1,TGM2,TNF,TREM1	cell movement of leukocytes

16	3.671	PI3K (complex)	19	Ccl2,CCL4,CCR1,CXCR4,FN1,IL12B,IL17A,IL1B,IL23A,IL6,MMP12,MMP9,PIK3CG,PTGS2,SELL,SPP1,TGM2,TNF,TREM1	cell movement of myeloid cells
17	3.671	Pam3-Cys-Ser-Lys4	19	CCL4,CCR7,CD83,CSF3,CXCL3,CXCL6,F3,IL12B,IL17A,IL1B,IL23A,IL2RA,IL6,LTB,MMP1,MMP9,PTGS2,SELP,TNF	activation of blood cells
18	3.618	FN1	11	CCL4,CXCL2,CXCL3,IL1B,IL6,ITGA1,MMP9,PLAUR,SPP1,TFF1,TNF	chemotaxis
19	3.618	FN1	11	CCL4,CXCL2,CXCL3,IL1B,IL6,ITGA1,MMP9,PLAUR,SPP1,TFF1,TNF	chemotaxis of cells
20	3.615	Salmonella enterica serotype abortus equi lipopolysaccharide	15	Ccl2,CCL3L3,CCL4,CCR7,CSF3,CXCL3,IL12B,IL1B,IL2RA,IL6,INHBA,NLRP3,PTGS2,TNF,WNT5A	cell movement of mononuclear leukocytes
21	3.615	diphenyleneiodonium	15	AGT,Ccl2,CXCL2,CXCL3,F3,FN1,IL1B,IL6,MMP12,MMP9,PTGS2,SELL,THBS2,TIMP1,TRPC6	cell movement of myeloid cells
22	3.615	uric acid	15	AGT,Ccl2,CCL3L3,CCL4,CSF3,CXCL2,CXCL3,IL12B,IL17A,IL1B,IL23A,IL6,PTGS2,TNF,TREM1	cell movement
23	3.615	uric acid	15	AGT,Ccl2,CCL3L3,CCL4,CSF3,CXCL2,CXCL3,IL12B,IL17A,IL1B,IL23A,IL6,PTGS2,TNF,TREM1	cell movement of blood cells
24	3.615	uric acid	15	AGT,Ccl2,CCL3L3,CCL4,CSF3,CXCL2,CXCL3,IL12B,IL17A,IL1B,IL23A,IL6,PTGS2,TNF,TREM1	cell movement of phagocytes
25	3.615	uric acid	15	AGT,Ccl2,CCL3L3,CCL4,CSF3,CXCL2,CXCL3,IL12B,IL17A,IL1B,IL23A,IL6,PTGS2,TNF,TREM1	inflammatory response
26	3.615	uric acid	15	AGT,Ccl2,CCL3L3,CCL4,CSF3,CXCL2,CXCL3,IL12B,IL17A,IL1B,IL23A,IL6,PTGS2,TNF,TREM1	migration of cells
27	3.606	Bay 11-7082	13	BCL2A1,BMP2,Ccl2,CCL3L3,CD83,CXCL2,F3,FPR2,IL17A,IL6,MMP9,PTGS2,TNF	generation of cells
28	3.606	C5AR1	13	C5,Ccl2,CSF3,CXCL2,CXCL3,IL12B,IL17A,IL17F,IL1B,IL23A,IL6,SELP,TNF	cell movement of blood cells
29	3.606	C5AR1	13	C5,Ccl2,CSF3,CXCL2,CXCL3,IL12B,IL17A,IL17F,IL1B,IL23A,IL6,SELP,TNF	leukocyte migration
30	3.606	SP600125	13	Ccl2,CCL4,CXCL3,FN1,IL17A,IL1B,IL23A,IL6,MMP9,PTGS2,SPP1,TNF,TNFSF11	activation of phagocytes
31	3.606	TNFSF12	13	Ccl2,CCL3L3,CCR1,CXCL2,CXCL3,CXCL6,IL17A,IL1B,IL6,MMP9,S100A8,S100A9,TNF	migration of neutrophils
32	3.606	diphenyleneiodonium	13	AGT,Ccl2,CXCL2,CXCL3,F3,FN1,IL1B,IL6,MMP9,PTGS2,SELL,THBS2,TRPC6	recruitment of cells
33	3.606	enterotoxin B	13	Ccl2,CCL3L3,CCL4,CSF3,CXCL2,CXCL3,CXCL6,CXCR4,IL12B,IL17A,IL1B,IL6,TNF	migration of phagocytes
34	3.606	poly rI:rC-RNA	13	Ccl2,CCL3L3,CCL4,CSF3,CXCL2,CXCL3,CXCR4,FPR1,IL11,IL17A,IL1B,TNF,TNFRSF11B	mobilization of blood cells

35	3.606	poly rI:rC-RNA	13	Ccl2,CCL3L3,CCL4,CSF3,CXCL2,CXCL3,CXCR4,FPR1,IL11,IL17A,IL1B,TNF,TNFRSF11B	mobilization of cells
36	3.606	trinitrobenzenesulfonic acid	13	Ccl2,CCL3L3,CCR1,CXCL2,CXCL3,CXCL6,IL17A,IL1B,IL6,MMP9,S100A8,S100A9,TNF	migration of neutrophils
37	3.578	MYD88	20	Ccl2,CCL3L3,CCL4,CXCL2,CXCL3,CXCL6,FPR1,FPR2,HP,IL12B,IL17A,IL1B,IL23A,INHBA,MMP7,S100A8,SAA1,SPP1,TNF,TNFSF11	chemotaxis of myeloid cells
38	3.5	E. coli lipopolysaccharide	16	Ccl2,CCL4,CCR7,CXCL2,CXCL3,CXCL6,IL12B,IL1B,IL23A,IL6,MMP9,S100A8,S100A9,TNF,TREM1,TREM3	cell movement of granulocytes
39	3.5	E. coli lipopolysaccharide	16	Ccl2,CCL4,CCR7,CXCL2,CXCL3,CXCL6,IL12B,IL1B,IL23A,IL6,MMP9,S100A8,S100A9,TNF,TREM1,TREM3	chemotaxis
40	3.5	E. coli lipopolysaccharide	16	Ccl2,CCL4,CCR7,CXCL2,CXCL3,CXCL6,IL12B,IL1B,IL23A,IL6,MMP9,S100A8,S100A9,TNF,TREM1,TREM3	chemotaxis of cells
41	3.5	FN1	16	CCNE1,CXCL2,F3,IL1B,IL6,ITGA1,MMP1,MMP13,MMP3,MMP9,PLAUR,RECK,RUNX2,SPP1,TGM2,TNF	angiogenesis
42	3.479	IL17A	10	Ccl2,CXCL2,CXCL3,CXCL6,IL1B,IL6,MMP9,S100A8,SELP,TNF	migration of neutrophils
43	3.479	IL23A	10	Ccl2,HP,ICOS,IL17A,IL17F,IL1B,IL6,S100A8,S100A9,TNF	leukocyte migration
44	3.474	Ap1	14	CCL3L3,CCL4,FN1,IL12B,IL1B,IL2RA,IL6,MMP12,MMP9,PTGS2,SPP1,TIMP1,TNF,TNFRSF8	cell movement of mononuclear leukocytes
45	3.474	ERK1/2	14	Ccl2,CCL3L3,CCL4,CCR7,CXCL3,CXCR4,F3,IL11,IL1B,IL6,PLAUR,PTGS2,SPP1,TNF	adhesion of immune cells
46	3.474	IL17RA	14	Ccl2,CCR1,CSF3,CSF3R,CXCL2,CXCL3,CXCL6,IL1B,IL6,MMP9,S100A8,S100A9,SELP,TNF	inflammatory response
47	3.474	uric acid	14	AGT,Ccl2,CCL3L3,CCL4,CSF3,CXCL2,CXCL3,IL12B,IL17A,IL1B,IL6,PTGS2,TNF,TREM1	cellular infiltration
48	3.464	C5AR1	12	C5,Ccl2,CSF3,CXCL2,CXCL3,IL12B,IL17A,IL1B,IL23A,IL6,SELP,TNF	homing of cells
49	3.464	ERK1/2	12	Ccl2,CCL3L3,CCL4,CXCL3,CXCL6,F3,IL1B,IL6,MMP9,PLAUR,SPP1,TNF	migration of granulocytes
50	3.464	ERK1/2	12	Ccl2,CCL3L3,CCL4,CXCL3,CXCL6,F3,IL1B,IL6,MMP9,PLAUR,SPP1,TNF	migration of myeloid cells

Supplementary Table 6. Upstream Regulators identified by IPA by comparing transcriptome of SA tumors with normal penis. Top 50 are shown. *P* values by Right-Tailed Fisher's Exact Test.

Upstream Regulator	Expr Log Ratio	Molecule Type	Predicted Activation State	Activation z-score	p-value of overlap
CTNNB1		transcription regulator	Activated	4.46	3.41E-28
IL1B	6.532	cytokine	Activated	4.51	1.99E-23
tretinoin		chemical - endogenous mammalian	Activated	2.572	4.47E-22
lipopolysaccharide		chemical drug	Activated	5.709	6.43E-22
beta-estradiol		chemical - endogenous mammalian	Activated	3.9	3.38E-21
IL10RA		transmembrane receptor	Inhibited	-4.473	5.27E-21
SOX2	7.099	transcription regulator		0.8	4.04E-20
TNF	3.33	cytokine	Activated	7.004	1.8E-19
phorbol myristate acetate		chemical drug	Activated	5.725	1.82E-19
IFNG		cytokine	Activated	3.861	4.17E-19
JUN		transcription regulator	Activated	4.709	3.55E-18
dexamethasone		chemical drug	Inhibited	-2.541	3.56E-18
KLF4		transcription regulator	Activated	3.923	8.67E-18
FOXA2	9.133	transcription regulator		-1.085	1.37E-16
trinitrobenzenesulfonic acid		chemical reagent	Activated	5.322	2.54E-16
Cg		complex	Activated	2.921	5.97E-16
ISL1		transcription regulator		1.39	3.93E-15
WNT3A		cytokine	Activated	3.499	7.28E-15
CEBPA		transcription regulator		1.085	1.07E-14
POU4F1		transcription regulator		0.772	1.1E-14
TGFB1		growth factor		1.774	6.01E-14
TNFSF12		cytokine	Activated	4.016	1.25E-13
Ca2+		chemical - endogenous mammalian	Activated	2.526	1.76E-13
mifepristone		chemical drug		1.067	1.9E-13
progesterone		chemical - endogenous mammalian		-0.432	5.51E-13
bucladesine		chemical toxicant	Activated	3.627	5.92E-13

NFkB (complex)		complex	Activated	4.139	6.04E-13
PD98059		chemical - kinase inhibitor	Inhibited	-4.61	8.13E-13
CEBPB		transcription regulator	Activated	4.674	8.66E-13
GATA4		transcription regulator		-1.358	9E-13
SHH	8.162	peptidase	Activated	4.524	1.03E-12
FGFR2		kinase		0.253	1.08E-12
IL13		cytokine		1.067	1.63E-12
GSTP1		enzyme			2.14E-12
LIF		cytokine		1.62	2.31E-12
STAT3		transcription regulator	Activated	3.019	2.56E-12
RELA		transcription regulator	Activated	3.335	2.65E-12
ESR2		ligand-dependent nuclear receptor		1.025	3.08E-12
decitabine		chemical drug	Activated	2.674	4.49E-12
EZH2		transcription regulator		-0.75	4.68E-12
forskolin		chemical toxicant	Activated	2.579	5.62E-12
NKX2-1		transcription regulator		0.673	9.7E-12
NOG		growth factor		-0.194	1.01E-11
SOX7		transcription regulator		-1.949	1.31E-11
U0126		chemical - kinase inhibitor	Inhibited	-4.621	1.41E-11
IL17RA		transmembrane receptor	Activated	3.939	1.97E-11
GLI2		transcription regulator	Activated	3.079	2.27E-11

Supplementary Table 7. Average of normalized RPPA values for mouse penile tumors of different genotypes and treatments.

Gene Name	Antibody Name	Antibody Origin	Wild type	APC	Pten	Smad4 APC	Smad4 APC + cisplatin	Pten Smad4 APC	Pten Smad4 APC + cisplatin
YWHAB	14-3-3-beta	R	1.12	1.18	1.00	0.87	0.98	0.90	0.91
YWHAE	14-3-3-epsilon	M	0.57	1.00	1.14	1.35	0.61	0.99	1.59
YWHAZ	14-3-3-zeta	R	1.01	0.97	0.95	0.96	1.07	1.08	0.91
EIF4EBP1	4E-BP1	R	0.73	0.72	0.75	1.13	0.91	1.16	1.29
EIF4EBP1	4E-BP1_pS65	R	1.16	1.06	0.84	0.93	0.90	0.88	0.87
TP53BP1	53BP1	R	1.03	0.98	0.92	1.06	1.32	1.05	0.89
ARAF	A-Raf	R	1.02	0.98	0.85	1.11	1.10	0.99	0.76
ACACA	ACC1	R	0.86	0.94	0.76	1.13	1.14	1.40	1.04
ACACA	ACC_pS79	R	0.78	0.77	0.65	1.08	1.26	1.17	1.18
ADAR	ADAR1	M	0.59	0.98	1.19	1.41	0.67	1.04	1.49
AKT1	Akt	R	1.11	0.91	0.86	1.05	1.10	0.91	0.61
AKT1	Akt_pS473	R	1.02	0.88	1.59	0.89	1.09	1.61	2.05
AKT1	Akt_pT308	R	1.07	0.92	1.14	0.88	0.94	1.11	1.43
PRKAA2	AMPK-a2_pS345	R	1.03	0.99	0.99	1.08	1.02	1.04	0.92
PRKAA1	AMPKa	R	1.12	1.09	0.93	0.97	1.00	0.96	0.84
PRKAA1	AMPKa_pT172	R	0.96	0.91	0.78	1.02	1.08	1.23	0.92
ANXA1	Annexin-I	M	0.44	0.88	1.33	1.31	0.57	0.95	1.57
ANXA7	Annexin-VII	M	0.63	1.08	1.16	1.66	0.72	1.11	1.57
AR	AR	R	1.94	1.45	1.30	0.92	0.62	0.62	0.50
ARID1A	ARID1A	R	0.99	1.02	0.95	1.13	1.33	1.27	0.91
ATG3	Atg3	R	1.01	1.00	0.94	0.88	0.96	0.96	0.88
ATG7	Atg7	R	0.98	1.01	0.97	1.08	1.21	1.02	0.93
ATM	ATM	R	0.97	0.96	0.89	1.06	1.14	1.00	0.84
ATM	ATM_pS1981	R	1.03	0.96	1.00	0.93	0.98	0.98	1.13
ATRX	ATRX	R	0.96	0.93	0.90	1.13	1.19	1.07	0.90
ATR	ATR_pS428	R	1.02	1.00	0.99	1.03	1.10	1.12	0.99
AIM1	Aurora-B	R	0.98	1.05	0.99	0.93	1.02	1.01	1.08
AXL	Axl	R	0.97	1.01	0.87	0.92	1.00	1.05	0.89
ACTB	b-Actin	R	0.96	0.99	0.82	1.28	0.96	0.92	1.06
CTNNB1	b-Catenin	R	1.20	1.12	0.87	0.89	1.17	1.15	1.02
CTNNB1	b- Catenin _pT41_S45	R	1.04	1.03	1.02	0.94	1.14	0.95	1.02
BRAF	B-Raf	R	1.06	1.02	0.88	0.96	0.93	0.92	1.00
BRAF	B-Raf_pS445	R	1.00	1.00	0.92	1.20	1.30	1.18	0.88
VTCN1	B7-H4	R	1.00	0.94	1.05	0.98	1.10	1.02	1.01
BAD	Bad_pS112	R	1.10	0.96	1.04	1.01	1.11	0.87	1.28
BAK1	Bak	R	1.04	1.03	1.03	0.94	0.97	0.95	1.13
BAP1	BAP1	M	0.55	0.95	1.27	1.31	0.60	0.93	1.50
BAX	Bax	R	0.86	0.88	0.92	1.11	1.24	1.06	1.04
BCL2L1	Bcl-xL	R	0.88	0.87	0.94	1.02	1.27	1.01	1.15
BCL2	Bcl2	M	1.31	2.28	1.39	1.25	0.54	1.29	2.06

BCL2A1	Bcl2A1	R	0.76	1.27	0.92	0.85	0.85	1.06	1.55
BECN1	Beclin	G	1.00	1.24	0.90	0.82	0.88	0.94	1.32
BID	Bid	R	1.01	0.97	0.90	1.04	1.03	1.07	1.09
BCL2L11	Bim	R	0.93	0.85	0.86	1.12	1.33	1.04	0.85
HSPA5	BiP-GRP78	M	0.48	1.02	1.19	1.34	0.45	0.92	1.33
BRD4	BRD4	R	0.98	0.96	0.90	0.96	1.03	1.04	0.91
ABL1	c-Abl	R	1.04	1.04	1.01	0.97	1.04	1.02	1.00
BIRC3	c-IAP2	R	0.98	0.98	1.01	1.01	1.00	1.04	0.90
JUN	c-Jun_pS73	R	1.06	1.03	1.06	0.94	1.15	1.09	1.05
KIT	c-Kit	R	1.05	1.02	1.04	1.04	1.03	1.07	0.97
MET	c-Met_pY1234_Y1235	R	1.04	0.99	0.92	1.01	1.01	1.04	0.93
MYC	c-Myc	R	0.92	1.03	0.86	0.90	1.00	1.04	1.33
RAF1	C-Raf	R	1.18	1.05	1.01	1.22	0.92	0.96	0.73
RAF1	C-Raf_pS338	R	1.08	1.02	0.93	1.10	1.10	1.06	0.99
CASP3	Caspase-3	R	0.98	0.98	0.94	1.03	0.98	1.04	0.89
CASP7	Caspase-7-cleaved	R	1.02	1.09	0.90	1.14	1.00	0.91	1.34
CAV1	Caveolin-1	R	5.14	2.80	3.19	0.98	0.65	0.64	0.44
L1CAM	CD171	M	0.51	0.98	1.35	1.45	0.57	0.99	1.70
DPP4	CD26	R	1.12	1.11	1.07	0.88	0.96	0.95	1.19
CD29	CD29	M	0.56	0.98	1.19	1.30	0.55	0.98	1.39
PECAM1	CD31	M	0.85	1.64	1.10	1.26	0.45	1.34	1.17
CD44	CD44	M	0.53	1.12	1.20	1.33	0.48	0.99	1.41
ITGA2	CD49b	M	0.56	1.07	1.17	1.37	0.71	1.14	1.51
CDC25C	cdc25C	R	1.00	0.96	0.99	0.99	1.00	1.01	0.95
CDK1	Cdc2_pY15	R	0.98	0.99	0.99	1.02	1.10	1.11	0.96
CDK1	CDK1	R	0.76	0.80	0.76	1.07	1.00	1.54	1.06
CHEK1	Chk1	M	0.53	0.91	1.61	1.57	0.62	1.02	1.56
CHEK1	Chk1_pS296	R	0.96	0.92	0.95	1.14	1.06	1.02	1.03
CHEK2	Chk2	M	0.51	0.98	1.19	1.43	0.59	0.95	1.57
CHEK2	Chk2_pT68	R	1.03	0.99	1.05	0.96	0.99	1.01	1.00
CLDN7	Claudin-7	R	1.04	0.93	0.94	1.10	0.90	0.96	1.12
COG3	COG3	R	0.92	1.03	0.94	0.93	0.96	0.99	1.26
COL6A1	Collagen-VI	R	1.53	1.39	1.12	0.88	1.08	0.88	0.94
CNST43	Connexin-43	R	0.51	1.15	0.61	0.83	1.12	1.49	1.24
PTGS3	Cox-IV	R	1.02	0.98	0.96	1.00	0.95	1.03	0.93
CMC2	Cox2	R	0.89	0.86	0.83	0.97	0.88	1.32	1.09
CREB1	Creb	R	1.01	0.91	0.96	0.98	0.99	0.95	1.55
CCNB1	Cyclin-B1	R	0.99	0.98	0.91	1.00	0.95	1.04	0.90
CCND1	Cyclin-D1	R	1.03	1.06	0.96	0.89	0.95	0.98	0.98
CCND3	Cyclin-D3	M	0.53	1.00	1.24	1.44	0.60	1.02	1.49
CCNE1	Cyclin-E1	M	0.53	1.01	1.15	1.31	0.59	0.99	1.49
PPIF	Cyclophilin-F	M	0.68	0.92	1.07	1.42	0.95	1.31	1.95
TUBA1A	D-a-Tubulin	R	1.02	0.83	0.83	0.95	1.00	1.14	1.29
PARK7	DJ1	R	1.12	0.99	1.10	1.00	1.14	0.92	0.92
HISTH3	DM-Histone-H3	R	0.92	1.20	0.83	0.85	0.91	1.02	1.23
H3K9ME2	DM-K9-Histone-H3	R	1.00	1.05	0.85	1.04	1.01	1.22	1.02

DUSP4	DUSP4	R	0.90	0.90	0.87	1.06	0.96	1.18	1.44
CDH1	E-Cadherin	R	1.01	0.97	1.01	1.00	1.09	0.94	0.90
E2F1	E2F1	M	0.59	1.05	1.19	1.40	0.64	1.07	1.53
EEF2	eEF2	R	0.76	0.90	0.73	1.03	1.06	1.39	1.09
EEF2K	eEF2K	R	1.24	1.27	1.17	0.85	0.90	0.96	1.24
EGFR	EGFR	R	1.04	0.98	0.97	0.99	1.11	1.04	1.15
EGFR	EGFR_pY1173	R	1.11	1.02	1.02	0.98	1.03	1.02	1.00
EIF4E	eIF4E	R	0.97	1.02	0.85	0.96	0.99	0.99	0.81
EIF4E	eIF4E_pS209	R	1.01	1.04	1.02	1.00	1.02	0.95	0.85
EIF4G1	eIF4G	R	1.02	1.06	0.96	1.06	1.16	1.36	0.79
ELK1	Elk1_pS383	R	1.01	0.99	0.91	0.90	1.00	0.97	1.12
EMA	EMA	M	0.48	0.93	1.31	1.31	0.51	0.88	1.62
ENY2	ENY2	M	0.51	0.94	1.40	1.39	0.55	0.93	1.42
ESR1	ER	R	1.04	0.99	1.05	1.04	1.09	1.03	0.97
ESR1	ER-a_pS118	R	1.08	1.02	1.01	1.09	1.02	1.06	0.91
ERCC1	ERCC1	M	0.70	1.08	1.15	1.35	0.79	1.11	1.51
ERCC5	ERCC5	R	0.85	1.36	0.87	0.94	0.95	1.00	1.98
ETS1	Ets-1	R	1.30	1.37	0.94	0.92	0.97	0.88	0.94
PTK2	FAK	R	1.36	1.54	0.97	0.97	1.07	0.90	0.75
PTK2	FAK_pY397	R	1.42	1.12	1.32	0.82	0.96	0.86	0.92
FASN	FASN	R	0.75	0.83	0.83	1.09	1.23	1.19	1.00
FN1	Fibronectin	R	0.95	0.94	0.50	0.97	0.62	1.03	1.16
FOXM1	FoxM1	R	1.06	0.96	0.99	0.92	0.97	0.95	0.97
FOX3	FoxO3a	R	1.04	1.00	1.02	0.94	0.98	0.99	0.94
FOXO3	FoxO3a_pS318_S321	R	1.00	1.08	0.95	0.89	0.95	0.90	1.36
FOSL1	FRA-1	R	1.05	1.04	0.95	0.98	1.13	0.99	1.04
G6PD	G6PD	R	0.95	0.96	0.85	0.90	0.99	1.14	1.57
GAB2	Gab2	R	1.29	1.00	1.10	0.95	1.17	1.00	0.58
GAPDH	GAPDH	M	0.56	0.90	0.73	1.24	0.68	0.98	1.23
GATA3	GATA3	M	0.70	1.02	1.24	1.39	0.67	0.93	1.49
GCLM	GCLM	R	1.04	1.01	1.00	0.98	0.93	1.04	0.94
KAT2A	GCN5L2	R	0.89	0.93	0.85	1.15	1.35	1.16	1.15
GLUD	Glutamate Dehydrogenase 1/2	R	1.27	1.09	1.15	0.90	0.91	0.90	0.86
GLS	Glutaminase	R	1.07	1.03	1.06	0.94	0.93	0.97	0.87
GZMB	Granzyme-B	R	1.15	1.05	1.19	0.90	0.97	0.92	0.94
GSK3A/GSK3B	GSK-3a-b	M	0.62	0.99	1.32	1.40	0.72	1.04	1.64
GSK3A GSK3B	GSK-3a-b_pS21_S9	R	1.27	1.17	1.07	0.95	1.27	0.81	1.05
GYS1	Gys	R	0.97	0.78	0.92	1.19	1.00	1.11	1.04
GYS1	Gys_pS641	R	1.21	0.98	1.11	1.11	1.13	0.89	0.92
H2AFX	H2AX_pS140	M	0.66	1.00	1.65	1.34	0.84	1.08	1.47
ERBB2	HER2	M	0.57	1.01	1.22	1.28	0.59	1.10	1.39
ERBB2	HER2_pY1248	R	1.26	1.29	1.14	0.83	1.12	0.93	0.99
ERBB3	HER3	R	1.09	1.00	0.87	0.95	1.06	1.06	0.82

ERBB3	HER3_pY1289	R	1.06	0.98	0.96	1.02	0.94	0.96	0.90
NRG1	Heregulin	R	0.87	1.05	0.84	0.85	1.13	1.25	1.56
HES1	HES1	R	1.01	1.00	1.04	1.26	1.37	1.20	0.89
HK2	Hexokinase-II	R	1.23	1.05	1.13	0.94	0.93	1.03	0.85
HIF1A	Hif-1-alpha	M	0.47	1.01	1.50	1.46	0.57	1.04	1.66
HIST3H3	Histone-H3	R	1.00	0.91	0.66	0.99	1.24	1.34	1.10
HSBP1	HSP27	M	0.49	0.95	1.19	1.39	0.56	0.91	1.48
HSBP1	HSP27_pS82	R	0.92	1.20	0.91	0.86	0.79	1.15	1.33
HSPA1A	HSP70	R	1.14	1.10	1.19	0.97	1.08	1.01	1.22
IGF1R	IGF1R_pY1135_Y1136	R	1.07	0.99	0.98	0.88	0.92	0.94	0.90
IGFBP2	IGFBP2	R	0.93	0.91	0.96	1.04	1.05	1.07	1.12
IGF1R	IGFRb	R	1.07	1.05	0.97	0.99	1.24	1.06	0.80
INPP4B	INPP4b	R	1.18	1.20	1.13	0.87	0.89	0.91	0.98
INSRB	IR-b	R	1.03	0.89	0.93	1.08	1.08	1.04	0.78
IRF1	IRF-1	R	1.95	1.96	1.43	0.88	1.06	0.68	0.69
IRS1	IRS1	R	1.02	0.99	1.01	1.02	1.11	1.09	1.15
JAG1	Jagged1	R	1.05	0.98	1.47	1.06	1.03	1.11	1.09
JAK2	Jak2	R	1.03	0.99	0.93	1.23	1.22	0.99	0.78
MAPK9	JNK2	R	1.15	1.00	1.01	0.99	1.09	0.94	0.88
MAPK8	JNK_pT183_Y185	R	1.08	1.00	1.02	0.99	1.05	1.01	1.03
LC3AB	LC3A-B	R	1.20	1.11	1.11	0.95	1.00	0.99	0.80
LCK	Lck	R	1.01	0.96	0.88	1.02	1.02	0.99	1.02
LDHA	LDHA	R	1.08	1.02	1.12	0.99	1.15	1.06	0.97
LRP6	LRP6_pS1490	R	1.03	1.04	1.03	0.92	1.07	1.01	0.95
MAPK3	MAPK_pT202_Y204	R	1.02	1.05	0.91	0.97	1.09	0.97	0.88
MCL1	Mcl-1	R	0.90	0.90	0.91	1.14	1.13	1.28	0.89
SLC16A4	MCT4	R	0.86	0.92	0.96	1.12	0.92	1.24	0.99
MDM2	MDM2_pS166	R	0.35	0.61	0.36	1.04	2.59	1.17	1.90
MAP2K1	MEK1	R	1.02	0.98	1.10	0.99	1.11	1.02	0.98
MAP2K1	MEK1_pS217_S221	R	1.05	0.99	1.02	1.12	1.28	1.02	0.98
BABAM1	MERIT40_pS29	R	0.90	1.07	1.01	0.92	1.02	1.01	0.88
NF2	Merlin	R	1.16	1.01	0.96	0.96	0.92	0.81	0.84
MIF	MIF	R	0.82	1.63	0.95	0.86	0.85	1.26	2.08
ERRFI1	MIG6	M	0.54	0.96	1.19	1.23	0.64	0.91	1.43
MMP2	MMP2	R	1.06	0.99	0.97	0.97	1.04	1.04	1.05
MKNK1	Mnk1	R	1.03	1.03	1.01	1.01	0.97	0.97	0.85
MSH6	MSH6	R	1.54	0.80	0.95	1.13	1.40	0.88	1.36
MSI2	MSI2	R	0.91	0.89	0.91	1.02	1.28	1.18	0.88
MTOR	mTOR	R	1.21	1.09	0.76	1.07	1.19	0.91	0.73
MTOR	mTOR_pS2448	R	0.91	0.98	0.70	1.25	1.52	0.83	1.74
MYH11	Myosin-11	R	2.40	2.44	1.68	0.86	0.49	0.76	0.86

MYO2A	Myosin-IIa_pS1943	R	1.00	1.05	1.09	1.08	1.16	1.21	0.96
MYT1	Myt1	R	0.85	0.87	0.89	0.91	0.93	1.05	1.16
CDH2	N-Cadherin	R	1.02	1.04	0.97	1.00	1.23	1.06	1.11
NRAS	N-Ras	M	0.56	0.90	1.21	1.39	0.78	1.08	1.46
NAPSA	NAPSIN-A	R	1.02	0.99	0.95	1.07	1.01	1.01	0.90
NDRG1	NDRG1_pT346	R	1.93	1.18	2.03	0.87	0.76	0.73	0.81
NDUFB4	NDUFB4	M	0.52	1.07	1.37	1.49	0.55	0.97	1.75
NFKB1	NF-kB-p65_pS536	R	1.05	0.99	0.89	1.03	1.00	0.98	1.00
NOTCH1	Notch1	R	1.16	1.15	0.99	0.87	1.01	1.19	1.03
NOTCH3	Notch3	R	1.03	1.13	1.04	0.87	0.98	1.02	1.26
OCT4	Oct-4	R	1.03	0.97	1.05	1.02	1.03	1.06	1.16
CDH3	P-Cadherin	R	0.47	0.76	0.63	1.27	1.22	1.13	1.09
CDKN2A	p16INK4a	R	0.71	0.84	0.89	1.17	1.55	1.13	0.94
CDKN1A	p21	R	1.04	0.92	0.93	0.96	0.98	1.05	0.98
CDKN1B	p27-Kip-1	R	1.07	1.01	1.00	1.03	1.02	1.01	0.93
CDKN1B	p27_pT198	R	1.03	0.98	0.93	0.92	0.99	1.00	1.21
MAPK14	p38-MAPK	R	1.00	0.96	0.77	1.08	0.98	0.96	0.88
MAPK14	p38_pT180_Y182	R	0.95	1.07	0.94	1.00	1.34	0.98	1.12
MAPK3	p44-42-MAPK	R	1.23	1.08	1.16	0.98	1.01	0.90	0.76
TP53	p53	R	0.92	1.03	0.96	0.89	0.95	1.03	1.98
RPS6KB1	p70-S6K1	R	1.15	1.06	0.98	1.03	1.12	0.90	0.87
RPS6KB1	p70-S6K_pT389	R	1.04	0.97	1.07	1.08	1.04	0.98	1.06
RPS6K	p90RSK_pT573	R	0.91	1.00	0.84	1.06	1.32	0.97	0.89
SERPINE1	PAI-1	M	0.48	0.91	1.24	1.41	0.62	0.95	1.40
PAICS	PAICS	R	1.07	0.99	0.99	1.01	1.10	1.06	0.85
PAK1	PAK1	R	0.83	0.79	0.80	1.36	1.34	1.36	0.94
PAK4	PAK4	R	1.06	0.96	1.06	0.90	1.01	0.96	1.18
PAR	PAR	R	0.50	0.58	0.48	1.07	1.04	1.08	1.18
PARP1	PARP1	R	0.81	1.19	0.84	1.06	0.95	0.97	1.59
PAX8	PAX8	R	0.95	0.87	0.93	0.97	0.96	1.03	1.13
PXN	Paxillin	R	1.25	0.98	1.03	0.96	1.25	0.99	0.79
PCNA	PCNA	M	0.62	1.19	1.36	1.52	0.54	1.02	1.90
CD274	PD-L1	R	1.07	1.01	1.13	0.94	1.04	1.02	1.03
PDCD1	Pdcd-1L1	G	1.17	1.62	1.25	0.77	0.94	0.83	1.40
PDCD4	Pdcd4	R	1.03	1.44	1.09	0.98	1.10	0.68	0.78
PDGFR	PDGFR-b	R	1.57	1.19	1.16	0.92	0.84	0.86	0.54
PDHK1	PDHK1	R	1.08	1.04	1.06	0.94	1.05	0.99	0.99
PDK1	PDK1	R	1.18	1.13	1.03	0.86	0.95	0.87	1.03
PDK1	PDK1_pS241	R	1.01	1.02	0.97	0.99	1.04	0.95	0.94
PEA15	PEA-15	R	1.10	1.00	1.06	1.01	1.02	0.94	0.89
PEA15	PEA-15_pS116	R	1.01	0.99	0.89	0.92	0.92	0.92	1.16
PIK3C2A	PI3K-p110-a	R	1.05	1.03	1.00	0.92	1.00	0.94	0.99
PIK3BC	PI3K-p110-b	M	0.79	1.00	1.19	1.51	0.77	1.18	1.58
PIK3R1	PI3K-p85	R	1.21	1.07	1.11	0.94	1.10	0.90	0.93

PRKAR1A	PKA-a	R	1.29	1.06	1.33	0.93	0.94	0.84	0.68
PRKCA	PKC-a_pS657	R	1.29	1.03	1.09	0.85	0.84	0.87	1.04
PRKCB	PKC-b-II_pS660	R	1.03	0.99	0.95	1.19	1.43	0.91	0.89
PRKCD	PKC-delta_pS664	R	0.89	3.89	1.19	1.10	0.88	0.86	3.91
PKM2	PKM2	R	0.56	0.67	0.67	1.15	0.94	1.60	1.22
PLCG2	PLC-gamma2_pY759	R	1.03	1.02	1.00	0.98	1.00	1.06	1.09
PLK1	PLK1	R	1.01	1.08	1.07	1.02	0.96	0.92	0.99
PMS2	PMS2	R	0.99	1.01	0.97	1.01	1.06	1.07	1.05
VDAC1	Porin	M	0.65	1.04	1.27	1.44	0.74	1.03	1.32
PGR	PR	R	0.99	0.97	0.97	1.06	1.00	1.08	0.98
AKT1S1	PRAS40	M	0.49	0.97	1.24	1.35	0.61	0.98	1.51
AKT1S1	PRAS40_pT246	R	1.11	0.99	0.98	0.98	0.97	0.87	1.04
PREX1	PREX1	R	0.94	1.05	1.03	0.94	0.92	1.01	1.08
PTEN	PTEN	R	1.21	1.07	0.96	1.25	1.19	0.60	0.46
RAB11A	Rab11	R	1.01	1.00	1.03	0.98	1.11	1.03	1.11
RAB25	Rab25	R	1.06	1.02	1.06	1.00	1.03	1.02	0.99
RAD50	Rad50	M	0.49	0.96	1.28	1.30	0.59	0.99	1.57
RAD51	Rad51	R	0.97	0.96	1.05	1.00	1.05	1.05	0.95
RPTOR	Raptor	R	1.14	1.12	0.92	1.01	1.08	0.93	0.86
RBM15	RBM15	R	1.13	1.30	1.39	0.86	1.09	0.86	0.83
RB1	Rb_pS807_S811	R	0.95	1.13	0.94	1.11	1.07	0.87	1.16
RHEB	Rheb	M	0.78	0.95	0.78	1.14	0.79	1.06	1.00
RICTOR	Rictor	R	3.71	3.77	2.94	0.80	0.82	0.87	0.70
RICTOR	Rictor_pT1135	R	1.16	1.06	0.92	0.88	0.97	0.91	1.02
RIP	RIP	R	1.03	1.06	1.24	0.97	1.11	1.06	0.92
ROCK1	Rock-1	R	1.21	1.07	0.86	0.86	0.80	0.72	1.10
RPA2	RPA32	T	0.97	0.94	1.02	1.18	1.20	1.11	1.62
RPA2	RPA32_pS4_S8	R	1.01	0.98	0.85	0.95	1.08	0.99	0.93
RPS6KA1	RSK	R	1.06	0.98	0.96	1.06	1.09	0.97	0.80
RPS6	S6	M	0.65	1.03	1.32	1.58	0.69	1.13	1.48
RPS6	S6_pS235_S236	R	0.85	1.11	1.08	1.51	1.14	0.86	0.78
RPS6	S6_pS240_S244	R	0.85	1.01	0.90	1.44	1.14	0.97	0.78
SCD	SCD	M	0.55	0.99	1.24	1.26	0.55	1.00	1.42
SDHA	SDHA	R	1.01	1.02	1.07	0.99	1.08	1.06	0.99
SRSF1	SF2	M	0.63	0.98	1.22	1.30	0.58	0.92	1.44
SHC1	Shc_pY317	R	1.08	1.04	1.01	0.90	1.05	1.01	0.94
PTPN11	SHP-2_pY542	R	1.21	1.05	1.06	0.91	0.95	0.89	0.85
SLC1A5	SLC1A5	R	0.88	0.84	0.85	1.19	1.14	1.35	0.89
SMAD1	Smad1	R	1.11	1.02	1.08	1.01	1.14	1.07	0.96

SMAD3	Smad3	R	1.06	1.01	0.86	0.97	0.96	0.92	0.93
SMAD4	Smad4	M	0.60	1.02	1.12	1.30	0.64	1.00	1.42
SOD1	SOD1	M	0.53	1.04	1.33	1.48	0.57	1.11	1.64
SOD2	SOD2	R	1.02	1.02	0.98	0.96	1.10	1.04	0.94
SOX2	Sox2	R	0.79	0.81	0.80	1.42	1.54	1.23	1.09
SRC	Src	M	0.55	1.03	1.11	1.40	0.72	1.08	1.48
SRC	Src_pY416	R	1.08	1.03	0.99	1.03	1.03	0.95	0.97
SRC	Src_pY527	R	1.48	1.16	1.36	0.86	0.99	0.58	0.60
STAT3	Stat3	R	1.11	0.96	0.95	1.15	1.12	0.90	0.69
STAT3	Stat3_pY705	R	0.94	0.91	0.91	1.36	1.39	1.04	0.95
STAT5A	Stat5a	R	1.63	1.63	1.35	0.87	1.05	0.89	0.71
STMN1	Stathmin-1	R	1.04	1.01	0.98	0.98	0.97	0.98	1.07
SYK	Syk	M	0.61	0.99	1.28	1.39	0.60	0.99	1.52
MAPT	Tau	M	0.51	0.98	1.29	1.34	0.62	1.01	1.60
TAZ	TAZ	R	1.10	0.92	0.81	1.02	0.95	0.90	1.08
TFAM	TFAM	R	0.97	1.01	1.01	0.96	1.04	1.05	1.21
TFRC	TFRC	R	0.99	0.97	1.10	1.06	1.29	1.21	1.05
TIGAR	TIGAR	R	1.16	1.05	0.87	1.00	0.93	0.92	1.06
TGM2	Transglutaminase	M	0.61	0.97	1.23	1.36	0.64	0.92	1.64
TRIM25	TRIM25	R	0.97	1.02	0.91	0.95	1.02	1.03	0.87
TSC1	TSC1	R	1.14	1.08	1.04	0.92	0.98	0.94	0.83
TTF1	TTF1	R	1.05	1.05	0.89	0.93	0.93	0.96	0.91
TSC2	Tuberin	R	1.07	0.97	0.96	1.08	1.01	0.98	0.88
TSC2	Tuberin_pT1462	R	1.55	1.60	1.06	0.91	1.06	0.91	0.87
TUFM	TUFM	R	1.04	0.98	1.08	1.06	1.05	1.06	0.93
TWIST1	TWIST	M	0.53	1.01	1.19	1.32	0.61	1.00	1.47
TYRO3	Tyro3	R	1.07	1.07	1.08	0.92	1.11	0.93	0.97
UBAC1	UBAC1	R	0.93	0.89	0.86	1.02	0.89	0.96	1.29
H2BFM	Ubq-Histone-H2B	M	0.51	1.00	1.29	1.42	0.61	1.03	1.56
UGT1A	UGT1A	M	0.68	0.98	1.19	1.36	0.83	1.10	1.31
ULK1	ULK1_pS757	R	1.02	0.98	0.92	0.99	1.05	1.04	0.90
VASP	VASP	R	0.75	0.81	0.79	0.91	0.86	1.09	1.49
KDR	VEGFR-2	R	1.04	0.90	0.91	1.08	1.04	1.12	0.91
EPPK1	VHL-EPPK1	M	0.53	0.96	1.39	1.32	0.56	0.94	1.74
VIM	Vimentin	M	0.51	0.94	1.20	1.32	0.59	0.92	1.48
WEE1	Wee1	R	1.00	0.98	1.06	0.96	1.08	1.05	1.03
WIPI1	WIPI1	R	0.83	1.09	0.72	1.11	1.00	1.07	1.15
WIPI2	WIPI2	R	1.06	1.01	1.00	0.95	1.00	1.03	0.96
XBP1	XBP-1	G	0.97	1.44	1.16	0.88	0.92	1.01	1.57
XPA	XPA	M	0.66	1.01	1.19	1.38	0.67	1.03	1.68
XPF	XPF	M	0.62	0.99	0.98	1.48	0.51	1.59	1.04
XRCC1	XRCC1	R	1.15	1.03	1.08	0.94	1.07	0.94	0.94
YAP1	YAP	R	0.97	1.05	0.92	0.90	0.99	1.01	1.30
YAP1	YAP_pS127	R	1.15	0.96	0.91	0.98	0.97	0.61	1.06
YBX1	YB1_pS102	R	1.10	0.94	1.05	0.94	0.90	0.90	1.34
ZAP70	ZAP-70	R	1.01	0.99	0.90	1.13	1.25	1.05	0.89

Supplementary Table 8. RPPA protein list for Venn diagram showing the overlap of SA vs. WT and SAP vs. WT proteomics. *P* values by two-sided Student's *t* test on normalized RPPA signals of WT (n = 5), SA (n = 8) and SAP (n = 4) samples.

Position in Venn Diagram	Protein ID	Gene name	Fold Change (SA/WT)	Fold Change (SAP/WT)	P value (SA vs. WT)	P value (SAP vs. WT)
1. Overlap protein (90 total)	MDM2_pS166	MDM2	2.996	3.371	0.001	0.000
1. Overlap protein (90 total)	P-Cadherin	CDH3	2.691	2.389	0.000	0.005
1. Overlap protein (90 total)	PAR	PAR	2.132	2.150	0.000	0.011
1. Overlap protein (90 total)	PKM2	PKM2	2.031	2.835	0.000	0.005
1. Overlap protein (90 total)	Sox2	SOX2	1.786	1.552	0.000	0.015
1. Overlap protein (90 total)	p16INK4a	CDKN2A	1.655	1.595	0.000	0.005
1. Overlap protein (90 total)	Connexin-43	CNST43	1.643	2.944	0.000	0.000
1. Overlap protein (90 total)	PAK1	PAK1	1.637	1.639	0.000	0.004
1. Overlap protein (90 total)	4E-BP1	EIF4EBP1	1.554	1.590	0.000	0.001
1. Overlap protein (90 total)	FASN	FASN	1.460	1.591	0.000	0.000
1. Overlap protein (90 total)	CDK1	CDK1	1.415	2.036	0.000	0.001
1. Overlap protein (90 total)	ACC_pS79	ACACA	1.393	1.515	0.001	0.019
1. Overlap protein (90 total)	eEF2	EEF2	1.366	1.837	0.000	0.008
1. Overlap protein (90 total)	SLC1A5	SLC1A5	1.354	1.537	0.000	0.000
1. Overlap protein (90 total)	WIPI1	WIPI1	1.328	1.279	0.000	0.004
1. Overlap protein (90 total)	MCT4	SLC16A4	1.314	1.451	0.000	0.000
1. Overlap protein (90 total)	ACC1	ACACA	1.311	1.625	0.000	0.002
1. Overlap protein (90 total)	PARP1	PARP1	1.306	1.195	0.000	0.005
1. Overlap protein (90 total)	Bax	BAX	1.293	1.229	0.000	0.000
1. Overlap protein (90 total)	GCN5L2	KAT2A	1.291	1.306	0.003	0.000
1. Overlap protein (90 total)	Mcl-1	MCL1	1.261	1.420	0.000	0.000
1. Overlap protein (90 total)	VASP	VASP	1.207	1.440	0.002	0.004
1. Overlap protein (90 total)	Bcl-xL	BCL2L1	1.152	1.140	0.000	0.026
1. Overlap protein (90 total)	ARID1A	ARID1A	1.132	1.277	0.043	0.000
1. Overlap protein (90 total)	Bcl2A1	BCL2A1	1.128	1.401	0.009	0.005
1. Overlap protein (90 total)	MSI2	MSI2	1.121	1.301	0.007	0.001
1. Overlap protein (90 total)	IGFBP2	IGFBP2	1.119	1.157	0.002	0.008
1. Overlap protein (90 total)	ERCC5	ERCC5	1.097	1.175	0.017	0.018
1. Overlap protein (90 total)	TAZ	TAZ	0.929	0.817	0.007	0.004
1. Overlap protein (90 total)	Bad_pS112	BAD	0.922	0.793	0.003	0.001
1. Overlap protein (90 total)	PEA-15	PEA15	0.921	0.858	0.015	0.001
1. Overlap protein (90 total)	Gys_pS641	GYS1	0.918	0.739	0.030	0.001
1. Overlap protein (90 total)	B-Raf	BRAF	0.914	0.872	0.031	0.037
1. Overlap protein (90 total)	PEA-15_pS116	PEA15	0.910	0.907	0.010	0.033
1. Overlap protein (90 total)	b-Catenin_pT41_S45	CTNNB1	0.903	0.910	0.005	0.025
1. Overlap protein (90 total)	ATM_pS1981	ATM	0.903	0.956	0.009	0.025
1. Overlap protein (90 total)	DJ1	PARK7	0.896	0.825	0.003	0.000
1. Overlap protein (90 total)	p70-S6K1	RPS6KB1	0.894	0.781	0.038	0.004
1. Overlap protein (90 total)	FoxO3a_pS318_S321	FOXO3	0.893	0.906	0.001	0.045
1. Overlap protein (90 total)	Raptor	RPTOR	0.891	0.815	0.001	0.000
1. Overlap protein (90 total)	mTOR	MTOR	0.886	0.754	0.036	0.003
1. Overlap protein (90 total)	PRAS40_pT246	AKT1S1	0.884	0.784	0.017	0.002

1. Overlap protein (90 total)	PI3K-p110-a	PIK3C2A	0.877	0.898	0.000	0.002
1. Overlap protein (90 total)	Akt_pS473	AKT1	0.875	1.585	0.041	0.035
1. Overlap protein (90 total)	AMPKa	PRKAA1	0.869	0.855	0.006	0.008
1. Overlap protein (90 total)	FoxM1	FOXM1	0.868	0.889	0.001	0.005
1. Overlap protein (90 total)	Tyro3	TYRO3	0.863	0.872	0.002	0.001
1. Overlap protein (90 total)	JNK2	MAPK9	0.859	0.820	0.000	0.000
1. Overlap protein (90 total)	YAP_pS127	YAP1	0.854	0.532	0.002	0.000
1. Overlap protein (90 total)	YB1_pS102	YBX1	0.854	0.820	0.000	0.000
1. Overlap protein (90 total)	Merlin	NF2	0.826	0.701	0.000	0.000
1. Overlap protein (90 total)	IGF1R_pY1135_Y1136	IGF1R	0.821	0.880	0.000	0.005
1. Overlap protein (90 total)	XRCC1	XRCC1	0.819	0.818	0.000	0.000
1. Overlap protein (90 total)	TSC1	TSC1	0.810	0.826	0.000	0.000
1. Overlap protein (90 total)	4E-BP1_pS65	EIF4EBP1	0.802	0.759	0.000	0.000
1. Overlap protein (90 total)	p44-42-MAPK	MAPK3	0.795	0.730	0.000	0.001
1. Overlap protein (90 total)	LC3A-B	LC3AB	0.788	0.826	0.000	0.008
1. Overlap protein (90 total)	Granzyme-B	GZMB	0.787	0.804	0.000	0.004
1. Overlap protein (90 total)	CD26	DPP4	0.786	0.852	0.000	0.007
1. Overlap protein (90 total)	PI3K-p85	PIK3R1	0.781	0.745	0.000	0.000
1. Overlap protein (90 total)	14-3-3-beta	YWHAB	0.778	0.803	0.000	0.002
1. Overlap protein (90 total)	Hexokinase-II	HK2	0.768	0.838	0.000	0.001
1. Overlap protein (90 total)	Paxillin	PXN	0.765	0.787	0.000	0.008
1. Overlap protein (90 total)	RBM15	RBM15	0.762	0.766	0.000	0.000
1. Overlap protein (90 total)	Rictor_pT1135	RICTOR	0.756	0.784	0.000	0.000
1. Overlap protein (90 total)	SHP-2_pY542	PTPN11	0.751	0.735	0.000	0.005
1. Overlap protein (90 total)	GSK-3a-b_pS21_S9	GSK3A GSK3B	0.743	0.634	0.000	0.001
1. Overlap protein (90 total)	Gab2	GAB2	0.735	0.774	0.000	0.001
1. Overlap protein (90 total)	PDK1	PDK1	0.733	0.737	0.000	0.000
1. Overlap protein (90 total)	INPP4b	INPP4B	0.732	0.767	0.000	0.002
1. Overlap protein (90 total)	PKA-a	PRKAR1A	0.719	0.651	0.000	0.000
1. Overlap protein (90 total)	Rock-1	ROCK1	0.713	0.597	0.002	0.002
1. Overlap protein (90 total)	FAK	PTK2	0.710	0.664	0.001	0.008
1. Overlap protein (90 total)	Glutamate-D1-2	GLUD	0.710	0.713	0.000	0.000
1. Overlap protein (90 total)	Ets-1	ETS1	0.704	0.675	0.000	0.007
1. Overlap protein (90 total)	eEF2K	EEF2K	0.685	0.776	0.000	0.000
1. Overlap protein (90 total)	HER2_pY1248	ERBB2	0.658	0.739	0.000	0.001
1. Overlap protein (90 total)	PKC-a_pS657	PRKCA	0.657	0.677	0.000	0.002
1. Overlap protein (90 total)	Tuberin_pT1462	TSC2	0.585	0.586	0.000	0.001
1. Overlap protein (90 total)	PDGFR-b	PDGFR	0.583	0.547	0.000	0.001
1. Overlap protein (90 total)	Src_pY527	SRC	0.583	0.394	0.000	0.000
1. Overlap protein (90 total)	Collagen-VI	COL6A1	0.575	0.576	0.000	0.001
1. Overlap protein (90 total)	FAK_pY397	PTK2	0.574	0.607	0.000	0.002
1. Overlap protein (90 total)	Stat5a	STAT5A	0.535	0.542	0.000	0.000
1. Overlap protein (90 total)	AR	AR	0.473	0.319	0.000	0.000
1. Overlap protein (90 total)	NDRG1_pT346	NDRG1	0.453	0.378	0.000	0.000
1. Overlap protein (90 total)	IRF-1	IRF1	0.452	0.349	0.000	0.001
1. Overlap protein (90 total)	Myosin-11	MYH11	0.359	0.316	0.000	0.001
1. Overlap protein (90 total)	Rictor	RICTOR	0.214	0.234	0.000	0.000
1. Overlap protein (90 total)	Caveolin-1	CAV1	0.190	0.124	0.000	0.000

2. WT vs. SA only (50 total)	S6_pS235_S236	RPS6	1.772	1.014	0.017	0.945
2. WT vs. SA only (50 total)	S6_pS240_S244	RPS6	1.689	1.138	0.011	0.510
2. WT vs. SA only (50 total)	Stat3_pY705	STAT3	1.449	1.105	0.000	0.339
2. WT vs. SA only (50 total)	b-Actin	ACTB	1.329	0.957	0.018	0.553
2. WT vs. SA only (50 total)	HES1	HES1	1.253	1.189	0.001	0.063
2. WT vs. SA only (50 total)	Gys	GYS1	1.227	1.137	0.015	0.097
2. WT vs. SA only (50 total)	B-Raf_pS445	BRAF	1.208	1.184	0.009	0.121
2. WT vs. SA only (50 total)	Bim	BCL2L11	1.205	1.113	0.000	0.067
2. WT vs. SA only (50 total)	Chk1_pS296	CHEK1	1.190	1.062	0.019	0.245
2. WT vs. SA only (50 total)	Jak2	JAK2	1.189	0.964	0.002	0.427
2. WT vs. SA only (50 total)	DUSP4	DUSP4	1.182	1.313	0.028	0.108
2. WT vs. SA only (50 total)	ATRX	ATRX	1.174	1.112	0.000	0.120
2. WT vs. SA only (50 total)	p90RSK_pT573	RPS6K	1.156	1.057	0.000	0.561
2. WT vs. SA only (50 total)	PKC-b-II_pS660	PRKCB	1.146	0.880	0.008	0.140
2. WT vs. SA only (50 total)	ZAP-70	ZAP70	1.121	1.037	0.032	0.329
2. WT vs. SA only (50 total)	ATM	ATM	1.102	1.035	0.014	0.472
2. WT vs. SA only (50 total)	Atg7	ATG7	1.099	1.044	0.025	0.275
2. WT vs. SA only (50 total)	IR-b	INSRB	1.056	1.017	0.044	0.742
2. WT vs. SA only (50 total)	EGFR	EGFR	0.951	1.005	0.012	0.886
2. WT vs. SA only (50 total)	MAPK_pT202_Y204	MAPK3	0.948	0.949	0.008	0.331
2. WT vs. SA only (50 total)	Stathmin-1	STMN1	0.946	0.949	0.025	0.147
2. WT vs. SA only (50 total)	SOD2	SOD2	0.943	1.020	0.020	0.557
2. WT vs. SA only (50 total)	Aurora-B	AIM1	0.942	1.029	0.049	0.504
2. WT vs. SA only (50 total)	Chk2_pT68	CHEK2	0.933	0.982	0.003	0.376
2. WT vs. SA only (50 total)	c-Abl	ABL1	0.932	0.985	0.009	0.631
2. WT vs. SA only (50 total)	FRA-1	FOSL1	0.930	0.946	0.030	0.311
2. WT vs. SA only (50 total)	p21	CDKN1A	0.923	1.013	0.015	0.662
2. WT vs. SA only (50 total)	JNK_pT183_Y185	MAPK8	0.916	0.934	0.000	0.070
2. WT vs. SA only (50 total)	Smad1	SMAD1	0.914	0.966	0.016	0.503
2. WT vs. SA only (50 total)	MMP2	MMP2	0.911	0.979	0.000	0.491
2. WT vs. SA only (50 total)	Bak	BAK1	0.905	0.912	0.032	0.119
2. WT vs. SA only (50 total)	FoxO3a	FOX3	0.904	0.950	0.005	0.251
2. WT vs. SA only (50 total)	WIPI2	WIPI2	0.896	0.969	0.005	0.505
2. WT vs. SA only (50 total)	Elk1_pS383	ELK1	0.893	0.960	0.006	0.240
2. WT vs. SA only (50 total)	LRP6_pS1490	LRP6	0.893	0.979	0.009	0.769
2. WT vs. SA only (50 total)	p27_pT198	CDKN1B	0.890	0.970	0.000	0.490
2. WT vs. SA only (50 total)	Glutaminase	GLS	0.887	0.913	0.002	0.051
2. WT vs. SA only (50 total)	EGFR_pY1173	EGFR	0.884	0.923	0.001	0.130
2. WT vs. SA only (50 total)	TTF1	TTF1	0.884	0.914	0.002	0.073
2. WT vs. SA only (50 total)	PD-L1	CD274	0.880	0.958	0.010	0.472
2. WT vs. SA only (50 total)	Atg3	ATG3	0.874	0.945	0.006	0.164
2. WT vs. SA only (50 total)	PDHK1	PDHK1	0.870	0.922	0.000	0.062
2. WT vs. SA only (50 total)	Cyclin-D1	CCND1	0.862	0.950	0.000	0.094
2. WT vs. SA only (50 total)	PAK4	PAK4	0.856	0.913	0.001	0.170
2. WT vs. SA only (50 total)	HSP70	HSPA1A	0.848	0.885	0.012	0.069
2. WT vs. SA only (50 total)	Notch3	NOTCH3	0.845	0.994	0.007	0.894
2. WT vs. SA only (50 total)	Shc_pY317	SHC1	0.838	0.938	0.001	0.290

2. WT vs. SA only (50 total)	Akt_pT308	AKT1	0.822	1.035	0.000	0.658
2. WT vs. SA only (50 total)	Notch1	NOTCH1	0.755	1.026	0.001	0.731
2. WT vs. SA only (50 total)	b-Catenin	CTNNB1	0.740	0.960	0.004	0.743
3. WT vs. SAP only (24 total)	MIF	MIF	1.047	1.527	0.533	0.017
3. WT vs. SAP only (24 total)	Cox2	CMC2	1.084	1.478	0.098	0.000
3. WT vs. SAP only (24 total)	eIF4G	EIF4G1	1.039	1.342	0.670	0.003
3. WT vs. SAP only (24 total)	Histone-H3	HIST3H3	0.984	1.336	0.862	0.020
3. WT vs. SAP only (24 total)	AMPKa_pT172	PRKAA1	1.059	1.275	0.479	0.022
3. WT vs. SAP only (24 total)	HSP27_pS82	HSBP1	0.939	1.250	0.282	0.025
3. WT vs. SAP only (24 total)	Myt1	MYT1	1.070	1.234	0.121	0.009
3. WT vs. SAP only (24 total)	DM-K9-Histone-H3	H3K9ME2	1.042	1.214	0.378	0.013
3. WT vs. SAP only (24 total)	Myosin-IIa_pS1943	MYO2A	1.082	1.208	0.092	0.008
3. WT vs. SAP only (24 total)	G6PD	G6PD	0.945	1.198	0.183	0.000
3. WT vs. SAP only (24 total)	c-Myc	MYC	0.979	1.133	0.698	0.036
3. WT vs. SAP only (24 total)	Cdc2_pY15	CDK1	1.045	1.131	0.211	0.014
3. WT vs. SAP only (24 total)	MERIT40_pS29	BABAM1	1.013	1.114	0.753	0.039
3. WT vs. SAP only (24 total)	DM-Histone-H3	HISTH3	0.929	1.110	0.073	0.030
3. WT vs. SAP only (24 total)	ATR_pS428	ATR	1.005	1.098	0.868	0.009
3. WT vs. SAP only (24 total)	COG3	COG3	1.012	1.082	0.668	0.033
3. WT vs. SAP only (24 total)	PMS2	PMS2	1.020	1.071	0.370	0.029
3. WT vs. SAP only (24 total)	Bid	BID	1.030	1.065	0.566	0.022
3. WT vs. SAP only (24 total)	N-Cadherin	CDH2	0.983	1.039	0.233	0.015
3. WT vs. SAP only (24 total)	Claudin-7	CLDN7	1.058	0.926	0.293	0.032
3. WT vs. SAP only (24 total)	Stat3	STAT3	1.031	0.812	0.315	0.008
3. WT vs. SAP only (24 total)	TIGAR	TIGAR	0.869	0.795	0.066	0.020
3. WT vs. SAP only (24 total)	Pdcd4	PDCD4	0.951	0.661	0.313	0.001
3. WT vs. SAP only (24 total)	PTEN	PTEN	1.032	0.494	0.367	0.000

Supplementary Table 9. Summary of IC50 for the inhibitor screen on SA1 and SAP1 cell lines.

		Cel line: SA1		Cell line: SAP1	
**Target**	**Drug**	**IC50 (μM)**	**95% CI**	**IC50 (μM)**	**95% CI**
PKC	Staurosporine	0.052	0.02859 to 0.09346	0.213	0.2052 to 0.2201
PI3K, mTOR	Dactolisib (BEZ235)	0.075	0.01981 to 0.2839	0.290	0.1721 to 0.4895
STAT3	LLL12	0.227	0.1870 to 0.2746	0.255	0.2328 to 0.2800
Chk1/2	AZD7762	0.231	0.2242 to 0.2381	2.732	2.274 to 3.282
Src	Dasatinib	0.282	0.1459 to 0.5464	0.861	0.6590 to 1.126
HDAC	Trichostatin A	0.525	0.4791 to 0.5744	0.413	0.1778 to 0.9605
mTOR	AZD8055	0.603	0.3900 to 0.9331	6.363	1.969 to 20.56
HER2	Mubritinib	0.909	0.3965 to 2.083	0.049	0.01589 to 0.1510
PI3K (p110α/β/δ/γ)	Buparlisib (BKM120)	1.347	0.8651 to 2.097	15.300	12.81 to 18.29
Microtubule	Paclitaxel	1.791	0.6953 to 4.616	6.498	3.444 to 12.26
CDK4/6	Palbociclib (PD0332991)	2.423	1.665 to 3.525	19.370	16.75 to 22.39
mTOR/PI3K	Vistusertib (AZD2014)	2.805	2.001 to 3.932	14.860	6.382 to 34.61
AKT1/2/3	MK-2206	3.066	1.852 to 5.077	2.871	2.065 to 3.991
AMPK	Dorsomorphin	3.138	2.033 to 4.843	28.460	16.17 to 50.08
HER2, EGFR	Lapatinib	3.496	1.807 to 6.763	20.110	15.36 to 26.33
Stat3	BP-1-102	3.965	0.9918 to 15.85	9.205	8.204 to 10.33
DNA synthesis	Cisplatin	4.568	3.366 to 6.200	29.740	23.21 to 38.12
MEK	PD325901	4.932	1.658 to 14.67	12.010	8.154 to 17.69
p70 S6K1	PF-4708671	5.694	4.325 to 7.495	5.420	4.301 to 6.829
Androgen Receptor	Enzalutamide	8.002	6.429 to 9.959	13.340	12.13 to 14.67
JAK2, STAT3	WP-1066 (TRC)	8.154	5.568 to 11.94	73.080	36.81 to 145.1
mTOR	Rapamycin	11.040	3.870 to 31.49	>100	>100
Bcl-xL, Bcl-2	Navitoclax (ABT-263)	11.900	9.324 to 15.19	4.982	1.844 to 13.46
VEGFR2, c-MET	Cabozantinib	13.690	10.24 to 18.30	30.830	14.33 to 66.34
Bcl-xL, Bcl-2	ABT-737	14.010	9.162 to 21.43	26.430	22.00 to 31.74
Chk1	MK-8776 (SCH 900776)	14.690	12.74 to 16.95	16.300	3.660 to 72.56
mTOR	Everolimus	16.510	11.70 to 23.29	>100	>100
BET bromodomain	(+)-JQ1	20.150	2.085 to 194.8	>100	>100
BET bromodomain	PFI-1	20.400	14.73 to 28.26	39.830	25.49 to 62.26
IGF-1R, InsR	BMS-754807	20.470	6.650 to 62.98	>100	>100
EGFR	Erlotinib	26.760	13.67 to 52.40	11.600	6.115 to 22.00
MEK1/2	UO126	27.270	19.15 to 38.85	41.870	15.88 to 110.5
JAK2	BMS-911543	31.250	15.84 to 61.66	18.040	16.09 to 20.23
JAK1/2	Ruxolitinib	32.800	20.86 to 51.58	>100	>100
DNA methylation	5-Azacytidine	36.930	31.69 to 43.04	>100	>100
DNA methylation	Decitabine	>100	>100	>100	>100
PI3Kα/δ	GDC-0941	>100	>100	>100	>100
Alkylating agent	Ifosfamide	>100	>100	>100	>100
mTOR	Ridaforolimus	>100	>100	>100	>100
γ secretase	RO4929097	>100	>100	>100	>100
STAT3	S3I-201	>100	>100	>100	>100
mTOR	Temsirolimus	>100	>100	>100	>100

Supplementary Table 10. List of upregulated and downregulated genes in human penile cancer relative to normal glan tissues (dataset from GSE57955), with their normalized expression values of homologous genes in SA and SAP tumors compared with wild type mouse penis.

Gene	Fold change (Human PeCa vs Normal)	Fold change (SAP vs. WT)	FDR (SAP vs. WT)	Fold change (SA vs. WT)	FDR (SA vs. WT)
MMP12	5.374	3.608	0.000	4.951	0.000
MMP10	3.140	5.368	0.000	9.203	0.000
IL1B	3.074	5.066	0.000	6.532	0.000
IL1A	2.561	1.922	0.000	1.474	0.000
CXCL1	2.322	2.419	0.002	2.417	0.000
EREG	1.628	-1.449	0.000	-1.249	0.000
CASC5	1.568	0.919	0.000	0.960	0.000
AIM2	1.558	2.130	0.000	2.179	0.000
RGS20	1.555	3.468	0.000	2.608	0.000
SKA3	1.384	1.113	0.001	0.790	0.001
CEP55	1.381	0.554	0.127	0.327	0.244
NETO2	1.304	2.686	0.000	4.221	0.000
BUB1	1.249	1.318	0.000	0.925	0.000
E2F7	1.239	1.236	0.000	1.124	0.000
SLC28A3	1.192	-0.299	0.183	-0.830	0.000
EPCAM	1.187	2.212	0.000	2.338	0.000
IL12RB2	1.182	-2.469	0.000	-2.251	0.000
CENPA	1.169	1.232	0.008	0.617	0.103
CXCL13	1.164	-4.394	0.000	1.086	0.386
CKAP2L	1.119	0.431	0.075	0.024	0.921
KRT16	1.117	1.252	0.000	1.199	0.000
TTK	1.071	1.362	0.000	0.992	0.000
CHEK1	1.042	0.105	0.762	-0.283	0.222
PKP1	1.011	-0.599	0.000	-1.162	0.000
MMP9	1.009	3.639	0.001	6.250	0.000
SHCBP1	0.992	1.198	0.000	0.728	0.001
NCAPG	0.953	1.266	0.000	0.859	0.000
IFNE	0.943	3.126	0.026	2.174	0.140
IL11	0.913	0.673	0.291	3.886	0.000
BNC1	0.904	0.501	0.038	0.653	0.001
IL1F5	0.897	-0.734	0.040	-0.719	0.000
EPGN	0.885	0.309	0.213	-0.609	0.001
AREG	0.858	1.611	0.000	0.936	0.002
MMP13	0.848	3.979	0.000	7.335	0.000
CYP24A1	0.848	1.195	NA	3.778	0.002
SPC25	0.847	0.790	0.013	0.276	0.316
SPRR2D	0.835	3.207	0.007	3.539	0.000
LCE3D	0.833	0.215	0.611	0.242	0.491
PPIF	0.828	0.111	0.677	-0.595	0.000
DLGAP5	0.814	0.707	0.057	0.502	0.080
HMMR	0.810	0.558	0.134	0.373	0.216
CCL20	0.807	1.103	0.474	2.123	0.043
CDK1	0.802	1.054	0.000	0.613	0.009
FOXM1	0.790	0.814	0.000	0.506	0.003

ERCC6L	0.789	0.804	0.010	0.632	0.005
BUB1B	0.787	0.823	0.002	0.370	0.092
HAS3	0.782	5.486	0.000	5.797	0.000
TNF	0.770	3.170	0.000	3.330	0.000
RAD51	0.765	0.598	0.011	0.173	0.411
KRT75	0.763	2.019	0.000	3.928	0.000
CYP27B1	0.759	0.938	0.238	0.300	0.687
LMNB1	0.754	0.862	0.000	0.636	0.000
MUCL1	0.744	-3.089	0.039	-3.389	0.008
TCHH	0.739	7.964	0.000	6.360	0.000
OVOL1	0.738	-0.461	0.042	-0.858	0.000
ASPM	0.732	0.289	0.462	0.312	0.298
TOP2A	0.724	0.847	0.000	0.514	0.007
KYNU	0.724	-0.930	0.580	1.103	0.280
KIF18A	0.720	0.514	0.103	0.431	0.075
GJB2	0.718	1.459	0.000	0.695	0.000
KIF20A	0.711	0.795	0.054	0.434	0.203
VSNL1	0.709	1.516	0.000	0.508	0.015
SERPINB5	0.708	-0.370	0.181	-0.876	0.000
UHRF1	0.707	0.888	0.000	0.520	0.000
FAM64A	0.704	0.904	0.026	0.325	0.340
TPX2	0.701	0.736	0.021	0.319	0.234
MELK	0.698	0.949	0.000	0.360	0.129
KRT17	0.684	3.015	0.000	2.587	0.000
MMP7	0.680	4.330	0.000	5.637	0.000
CCNA2	0.678	0.702	0.014	0.252	0.296
SLC7A11	0.662	-1.001	0.000	-0.847	0.000
KRT33A	0.655	2.376	0.000	1.643	0.000
NUF2	0.653	0.652	0.067	0.221	0.441
CENPF	0.649	0.521	0.177	0.310	0.306
MCM10	0.649	1.462	0.000	1.170	0.000
DTL	0.647	0.864	0.000	0.338	0.130
PBK	0.646	1.232	0.000	0.582	0.017
CDCA5	0.644	1.250	0.000	0.798	0.005
UBE2T	0.644	0.509	0.282	0.286	0.439
GAL	0.644	4.296	0.000	5.298	0.000
CDC6	0.644	0.497	0.112	0.083	0.743
CDCA8	0.640	1.000	0.002	0.422	0.127
CCNB1	0.639	1.043	0.003	0.488	0.087
AGMAT	0.633	4.183	0.000	4.674	0.000
TNNT1	0.623	-3.946	0.000	-4.141	0.000
B3GNT5	0.620	0.827	0.417	2.194	0.000
HSPB2	-1.494	-0.821	0.203	-0.254	0.569
FHL5	-1.500	-1.151	0.335	0.368	0.696
MAGI2	-1.500	-0.091	0.850	1.258	0.000
MAL	-1.512	1.031	0.018	1.381	0.000
HSPB7	-1.530	-3.756	0.000	-2.517	0.003
SORBS1	-1.530	-1.564	0.000	-1.660	0.000
PKNOX2	-1.542	-3.644	0.000	-3.434	0.000
FOXF1	-1.548	0.743	0.243	1.865	0.000

PNMT	-1.626	-0.100	NA	0.134	NA
CCDC85A	-1.638	-2.626	0.004	-2.045	0.003
MYLK	-1.644	-3.664	0.000	-3.328	0.000
THBS4	-1.674	-6.628	0.000	-3.658	0.000
PLCB4	-1.686	-1.645	0.000	-0.582	0.031
RAI2	-1.698	-3.137	0.000	-1.598	0.000
SRD5A2	-1.733	-3.620	0.000	-1.902	0.003
ITIH3	-1.733	-1.092	0.250	-0.410	0.648
SLITRK4	-1.739	0.212	NA	1.243	NA
TMOD1	-1.745	-2.179	0.000	-2.431	0.000
MAOB	-1.751	-4.030	0.000	-2.620	0.001
BEX1	-1.775	6.921	0.000	6.751	0.000
AR	-1.823	-2.167	0.000	-2.128	0.000
PRUNE2	-1.841	-0.481	0.387	1.211	0.000
XKR4	-1.847	-0.657	0.323	-1.459	0.003
TMEM47	-1.865	-0.561	0.121	1.104	0.000
CLEC3B	-1.877	-5.240	0.000	-4.060	0.000
CRLF1	-1.907	-2.439	0.000	-0.211	0.152
NLGN1	-1.955	0.196	0.818	1.785	0.000
NUDT10	-1.961	1.087	0.085	1.809	0.000
SCARA5	-1.973	-2.442	0.025	0.250	0.788
ZBTB16	-1.979	-4.134	0.000	-4.531	0.000
KCNB1	-1.990	-2.557	0.000	-2.254	0.000
CNR1	-1.990	-0.930	0.145	0.647	0.294
PTCHD1	-2.002	-2.987	0.000	-1.136	0.068
FRY	-2.008	-0.675	0.001	-0.177	0.321
GPM6A	-2.038	-3.085	0.000	-2.346	0.000
LMOD1	-2.044	-3.935	0.000	-4.255	0.000
KCNE4	-2.044	-1.970	0.000	-1.722	0.000
MYH11	-2.086	-4.861	0.000	-4.566	0.000
PDK4	-2.134	-0.208	0.635	0.789	0.000
NTF3	-2.140	-1.011	0.019	-0.516	0.121
GRIN2A	-2.224	-1.385	0.305	0.275	0.799
CASQ2	-2.283	-0.664	0.049	-1.907	0.000
PPP1R3C	-2.313	-3.758	0.000	-2.835	0.000
TMEM100	-2.331	-2.954	0.000	-0.360	0.222
PLN	-2.379	-5.217	0.000	-2.373	0.000
PPP1R1A	-2.433	2.873	0.000	3.742	0.000
RNF112	-2.451	-1.024	0.192	-0.938	0.145
FHL1	-2.469	-3.384	0.000	-2.091	0.000
PDZRN4	-2.487	-2.411	0.000	-2.144	0.000
TYRP1	-2.558	-3.818	0.006	-3.977	0.000
ARHGAP6	-2.606	-3.461	0.000	-1.305	0.000
GNAZ	-2.642	-1.493	0.007	-0.619	0.084
PDLIM3	-2.666	-2.221	0.000	-3.893	0.000
SCRG1	-2.917	0.635	0.685	-2.019	0.190
ARHGAP20	-2.947	-1.793	0.001	-0.245	0.626
LPHN3	-2.965	2.102	0.000	3.700	0.000
DES	-2.971	-4.835	0.000	-4.653	0.000
IPO9	-2.971	0.530	0.001	0.507	0.000

SPARCL1	-2.977	-1.629	0.000	-0.754	0.028
MYOT	-3.078	-0.577	NA	1.720	0.220
SYNM	-3.114	-3.662	0.000	-2.476	0.000
LONRF2	-3.144	0.202	0.778	-0.975	0.065
ITGA8	-3.150	-4.407	0.000	-3.568	0.000
SLC17A8	-3.162	-1.034	0.411	-0.986	0.262
PLP1	-3.228	-2.243	0.000	-0.130	0.739
ADRA1B	-3.431	-1.613	0.001	-1.079	0.004
PRDM16	-3.545	-1.082	0.001	-0.996	0.000
EDN3	-3.604	-4.715	0.000	-2.807	0.000
RCAN2	-3.604	-1.266	0.006	0.189	0.454
RGS5	-3.700	-0.285	0.560	0.973	0.003
ACTG2	-3.754	-4.849	0.000	-4.722	0.000
VIT	-3.855	-2.411	0.000	-0.461	0.072
PLCXD3	-3.915	-2.098	0.073	-1.151	0.126
OLFM4	-3.957	2.534	0.057	2.026	0.103
CILP	-4.160	-4.144	0.000	0.366	0.717
FAM150B	-4.160	0.040	0.979	-2.351	0.071
PCOLCE2	-4.196	-4.406	0.000	-4.357	0.000
MYRIP	-4.513	-1.576	0.000	-2.520	0.000
AOC3	-4.949	-2.988	0.000	-2.187	0.000
PCDH20	-5.320	0.879	0.024	0.190	0.551
CHRDL1	-5.326	-4.685	0.000	-2.134	0.027
CNN1	-5.912	-5.267	0.000	-4.846	0.000
MYOC	-8.195	-8.913	0.000	-9.148	0.000
FRZB	-11.626	1.113	0.177	3.615	0.000
ATP1A2	-14.728	1.156	0.333	1.964	0.003
ANGPTL7	-20.777	-5.953	0.000	-4.956	0.000

Supplementary Table 11. Enriched canonical pathways in human penile cancer samples relative to normal glans by IPA analysis of dataset GSE57955. *P* values by Right-Tailed Fisher's Exact Test.

Ingenuity Canonical Pathways	P value	Ratio	z-score
Agranulocyte Adhesion and Diapedesis	7.07946E-09	0.285	#NUM!
Cellular Effects of Sildenafil (Viagra)	6.45654E-08	0.302	#NUM!
Hepatic Fibrosis / Hepatic Stellate Cell Activation	1.1749E-07	0.265	#NUM!
Osteoarthritis Pathway	2.13796E-07	0.254	1.206
Gap Junction Signaling	2.18776E-07	0.258	#NUM!
Adrenomedullin signaling pathway	2.45471E-06	0.245	-3.727
Role of IL-17A in Psoriasis	2.69153E-06	0.692	#NUM!
Axonal Guidance Signaling	4.57088E-06	0.196	#NUM!
STAT3 Pathway	8.70964E-06	0.289	-1.512
Granulocyte Adhesion and Diapedesis	8.70964E-06	0.247	#NUM!
RhoGDI Signaling	1.09648E-05	0.24	2
Endothelin-1 Signaling	1.31826E-05	0.236	-1.852
CXCR4 Signaling	2.39883E-05	0.239	-3.087
G-Protein Coupled Receptor Signaling	3.46737E-05	0.208	#NUM!
Sertoli Cell-Sertoli Cell Junction Signaling	5.7544E-05	0.231	#NUM!
Thrombin Signaling	6.76083E-05	0.22	-1.768
Aryl Hydrocarbon Receptor Signaling	7.4131E-05	0.243	1.069
Signaling by Rho Family GTPases	7.58578E-05	0.208	-2.832
Role of Osteoblasts, Osteoclasts and Chondrocytes in Rheumatoid Arthritis	0.000102329	0.211	#NUM!
Sperm Motility	0.000112202	0.25	-3.78
Cardiac β-adrenergic Signaling	0.000134896	0.236	-2.887
Germ Cell-Sertoli Cell Junction Signaling	0.000239883	0.22	#NUM!
Tec Kinase Signaling	0.000239883	0.222	-2.502
Colorectal Cancer Metastasis Signaling	0.000263027	0.202	-1.857
Glutathione-mediated Detoxification	0.000281838	0.417	-2.53
Molecular Mechanisms of Cancer	0.000398107	0.182	#NUM!
Epithelial Adherens Junction Signaling	0.000398107	0.225	#NUM!
Relaxin Signaling	0.000398107	0.221	-3.9
Glioblastoma Multiforme Signaling	0.000446684	0.217	-3.182
Nitric Oxide Signaling in the Cardiovascular System	0.000467735	0.241	-3.674
Differential Regulation of Cytokine Production in Intestinal Epithelial Cells by IL-17A and IL-17F	0.000660693	0.409	#NUM!
Role of Macrophages, Fibroblasts and Endothelial Cells in Rheumatoid Arthritis	0.000691831	0.187	#NUM!
Breast Cancer Regulation by Stathmin1	0.000758578	0.202	#NUM!
Agrin Interactions at Neuromuscular Junction	0.000870964	0.269	-1.291
Cardiac Hypertrophy Signaling	0.000891251	0.196	-4.629
ILK Signaling	0.000912011	0.203	-3.333
Wnt/β-catenin Signaling	0.001023293	0.208	1.3
LPS/IL-1 Mediated Inhibition of RXR Function	0.001047129	0.199	1.265
Airway Pathology in Chronic Obstructive Pulmonary Disease	0.001096478	0.625	#NUM!
Renin-Angiotensin Signaling	0.00128825	0.223	-2.837
Regulation of Actin-based Motility by Rho	0.00128825	0.244	-1.789
Inhibition of Matrix Metalloproteases	0.001348963	0.316	-3.317
CDK5 Signaling	0.001412538	0.235	-1.706

14-3-3-mediated Signaling	0.00144544	0.219	-1.877
Glioma Invasiveness Signaling	0.001513561	0.257	-0.471
Glucocorticoid Receptor Signaling	0.001778279	0.178	#NUM!
Ovarian Cancer Signaling	0.001778279	0.211	-2.324
Cholecystokinin/Gastrin-mediated Signaling	0.001862087	0.23	-1.043
Protein Kinase A Signaling	0.001905461	0.174	-3.474
Cell Cycle: G2/M DNA Damage Checkpoint Regulation	0.002041738	0.28	-1.387
Synaptic Long Term Depression	0.002290868	0.202	-3.656
Regulation of the Epithelial-Mesenchymal Transition Pathway	0.002511886	0.197	#NUM!
Human Embryonic Stem Cell Pluripotency	0.002570396	0.209	#NUM!
Hepatic Cholestasis	0.002630268	0.203	#NUM!
Leukocyte Extravasation Signaling	0.002884032	0.191	-0.169
Sphingosine-1-phosphate Signaling	0.003090295	0.213	-2.353
Mitotic Roles of Polo-Like Kinase	0.003090295	0.254	2.309
Inhibition of Angiogenesis by TSP1	0.003630781	0.312	0.707
Synaptic Long Term Potentiation	0.003981072	0.212	-2.449
HMGB1 Signaling	0.004168694	0.206	-0.784
P2Y Purigenic Receptor Signaling Pathway	0.004677351	0.205	-4.041
Factors Promoting Cardiogenesis in Vertebrates	0.004677351	0.225	#NUM!
cAMP-mediated signaling	0.004677351	0.184	-4.323
Role of Tissue Factor in Cancer	0.005011872	0.208	#NUM!
GPCR-Mediated Integration of Enteroendocrine Signaling Exemplified by an L Cell	0.005248075	0.236	-0.728
Atherosclerosis Signaling	0.005495409	0.207	#NUM!
Thyroid Cancer Signaling	0.005623413	0.282	#NUM!
Dopamine-DARPP32 Feedback in cAMP Signaling	0.006025596	0.194	-3.9
Melanocyte Development and Pigmentation Signaling	0.006025596	0.216	-3.153
Neuregulin Signaling	0.00616595	0.224	-1.414
α-Adrenergic Signaling	0.00616595	0.224	-2.111
Virus Entry via Endocytic Pathways	0.006606934	0.209	#NUM!
GPCR-Mediated Nutrient Sensing in Enteroendocrine Cells	0.006606934	0.209	-3.962
Noradrenaline and Adrenaline Degradation	0.007413102	0.286	-1.897
Role of IL-17F in Allergic Inflammatory Airway Diseases	0.00851138	0.268	2.333
eNOS Signaling	0.008709636	0.189	-3.8
RAR Activation	0.008709636	0.185	#NUM!
Retinoate Biosynthesis I	0.009772372	0.29	-1
Role of NANOG in Mammalian Embryonic Stem Cell Pluripotency	0.009772372	0.2	-2.53
Gαq Signaling	0.01	0.189	-3.651
Estrogen-mediated S-phase Entry	0.01	0.308	2.121
tRNA Splicing	0.01023293	0.262	-2.714
Salvage Pathways of Pyrimidine Deoxyribonucleotides	0.01023293	0.5	2
Corticotropin Releasing Hormone Signaling	0.010964782	0.194	-2.858
Actin Cytoskeleton Signaling	0.011220185	0.176	-1.826
Role of Pattern Recognition Receptors in Recognition of Bacteria and Viruses	0.011481536	0.195	-1.387
CREB Signaling in Neurons	0.011748976	0.178	-4.158
Ethanol Degradation II	0.012022644	0.281	-1.667
ErbB Signaling	0.012589254	0.206	-1.342

Paxillin Signaling	0.012882496	0.2	-2.668
Leptin Signaling in Obesity	0.013182567	0.212	-2.646
Estrogen Biosynthesis	0.013489629	0.263	-2.53
Mouse Embryonic Stem Cell Pluripotency	0.015135612	0.2	-3.273
Creatine-phosphate Biosynthesis	0.015135612	0.6	#NUM!
Integrin Signaling	0.015848932	0.175	-3.182
PXR/RXR Activation	0.015848932	0.226	#NUM!
Bladder Cancer Signaling	0.016595869	0.207	-1.89
PAK Signaling	0.017378008	0.2	-2.524
3-phosphoinositide Biosynthesis	0.017378008	0.176	-2.744
Role of IL-17A in Arthritis	0.017782794	0.217	#NUM!
Role of NFAT in Cardiac Hypertrophy	0.019498446	0.172	-4.439
Gαs Signaling	0.020417379	0.194	-3.71
IL-6 Signaling	0.020892961	0.188	-0.816
Bupropion Degradation	0.021379621	0.292	-2.646
IL-8 Signaling	0.021877616	0.173	-2.611
Coagulation System	0.021877616	0.257	1
DNA damage-induced 14-3-3σ Signaling	0.021877616	0.316	#NUM!
GP6 Signaling Pathway	0.022908677	0.186	-2.449
Mechanisms of Viral Exit from Host Cells	0.022908677	0.244	#NUM!
HIF1α Signaling	0.023442288	0.19	#NUM!
Remodeling of Epithelial Adherens Junctions	0.023442288	0.215	#NUM!
Xenobiotic Metabolism Signaling	0.023442288	0.165	#NUM!
Tight Junction Signaling	0.025703958	0.177	#NUM!
IL-1 Signaling	0.025703958	0.198	-1.5
Role of Hypercytokinemia/hyperchemokinemia in the Pathogenesis of Influenza	0.02630268	0.25	#NUM!
IL-17A Signaling in Gastric Cells	0.02630268	0.28	1.342
Superpathway of Inositol Phosphate Compounds	0.026915348	0.167	-3.244
Role of Cytokines in Mediating Communication between Immune Cells	0.027542287	0.229	#NUM!
Phagosome Formation	0.030199517	0.185	#NUM!
Cell Cycle Regulation by BTG Family Proteins	0.030902954	0.243	2
Neuropathic Pain Signaling In Dorsal Horn Neurons	0.032359366	0.186	-4.146
Gustation Pathway	0.033113112	0.177	#NUM!
Phospholipase C Signaling	0.033884416	0.165	-2.828
Gα12/13 Signaling	0.034673685	0.179	-2.449
Eicosanoid Signaling	0.034673685	0.21	-0.816
Ethanol Degradation IV	0.035481339	0.286	-2.449
Nicotine Degradation II	0.036307805	0.22	-3.317
Nicotine Degradation III	0.036307805	0.227	-3.162
Basal Cell Carcinoma Signaling	0.037153523	0.203	-0.302
p38 MAPK Signaling	0.03801894	0.183	0.471
RhoA Signaling	0.038904514	0.18	-1.964
Role of CHK Proteins in Cell Cycle Checkpoint Control	0.039810717	0.211	-1.897
Melatonin Signaling	0.041686938	0.2	-3.051
Myc Mediated Apoptosis Signaling	0.041686938	0.2	#NUM!
Opioid Signaling Pathway	0.042657952	0.162	-3
Tryptophan Degradation to 2-amino-3-carboxymuconate Semialdehyde	0.043651583	0.429	#NUM!

Production of Nitric Oxide and Reactive Oxygen Species in Macrophages	0.044668359	0.166	-1.061
ATM Signaling	0.045708819	0.186	0.258
p70S6K Signaling	0.045708819	0.176	-2.985
Growth Hormone Signaling	0.045708819	0.19	-1.604
Acetone Degradation I (to Methylglyoxal)	0.046773514	0.25	-2.646
Differential Regulation of Cytokine Production in Macrophages and T Helper Cells by IL-17A and IL-17F	0.047863009	0.294	#NUM!
Oxidative Ethanol Degradation III	0.047863009	0.294	-2.236
Oncostatin M Signaling	0.048977882	0.235	0.707
Netrin Signaling	0.048977882	0.2	#NUM!
FXR/RXR Activation	0.048977882	0.176	#NUM!

Supplementary Table 12. Upstream Regulators identified by IPA by comparing transcriptome of human penile tumors compared with normal glans. Top 50 are shown. *P* values by Right-Tailed Fisher's Exact Test.

Upstream Regulator	Expr Log Ratio	Molecule Type	Predicted Activation State	Activation z-score	p-value of overlap
beta-estradiol		chemical - endogenous mammalian		-1.276	5.3E-36
TGFB1	0.904	growth factor	Inhibited	-2.019	2.12E-31
ERBB2	-0.469	kinase	Activated	5.723	9.72E-31
Vegf		group		0.956	1.62E-27
HGF	-3.289	growth factor	Activated	2.524	1.04E-23
progesterone		chemical - endogenous mammalian	Inhibited	-2.37	1.38E-23
dextran sulfate		chemical drug		1.231	1.46E-21
TNF	3.683	cytokine	Activated	5.585	3.15E-21
dexamethasone		chemical drug	Inhibited	-6.767	9.19E-20
TP53	0.597	transcription regulator	Inhibited	-3.566	1.19E-19
CCND1	-0.251	transcription regulator	Activated	4.218	2.52E-19
PTGER2	-2.006	g-protein coupled receptor	Activated	5.143	1.34E-18
IL1B	5.625	cytokine	Activated	3.977	3.24E-18
CTNNB1	-1.085	transcription regulator	Inhibited	-2.698	5.26E-18
phorbol myristate acetate		chemical drug	Activated	3.014	1.23E-17
ESR2	0.37	ligand-dependent nuclear receptor		-0.658	1.49E-17
CDKN1A	0.854	kinase	Inhibited	-3.599	1.69E-17
SMARCA4	1.649	transcription regulator	Inhibited	-3.254	3.97E-17
LY294002		chemical - kinase inhibitor		-0.033	5.32E-17
calcitriol		chemical drug	Inhibited	-3.539	5.9E-17
lipopolysaccharide		chemical drug	Activated	5.235	1.25E-16
ESR1	-2.911	ligand-dependent nuclear receptor		0.178	2.3E-16
SP1	0.354	transcription regulator		-1.349	2.3E-16
IFNG	2.418	cytokine	Activated	4.124	2.83E-16
Cg		complex		-1.49	2.33E-15
S100A8	2.21	other	Activated	3.073	2.53E-15
PD98059		chemical - kinase inhibitor		-1.903	3.16E-15
IL6	0.439	cytokine		-0.019	3.35E-15
Irgm1		other	Inhibited	-4.199	5.58E-15
IL17A	-0.222	cytokine	Activated	2.034	9.39E-15
AR	-4.758	ligand-dependent nuclear receptor		-1.143	1.37E-14
DNMT3B	1.509	enzyme	Activated	3.88	1.93E-14
S100A9	2.156	other	Activated	3.236	2.72E-14
trichostatin A		chemical drug		-1.218	3.48E-14
CSF2	2.404	cytokine	Activated	5.515	6.54E-14
F2	0.602	peptidase		-1.048	2.15E-13
JUN	-1.633	transcription regulator	Activated	2.876	2.18E-13
forskolin		chemical toxicant	Inhibited	-2.571	2.42E-13
MEF2C	-2.812	transcription regulator	Inhibited	-3.335	3.07E-13

gefitinib		chemical drug		-0.126	7.14E-13
tretinoin		chemical - endogenous mammalian	Inhibited	-3.089	9.81E-13
HNRNPA2B1	0.549	other		-0.293	1.14E-12
mifepristone		chemical drug	Activated	3.393	1.55E-12
P38 MAPK		group		0.744	2.56E-12
cycloheximide		chemical reagent		0.088	3.08E-12
decitabine		chemical drug		-1.159	3.4E-12
EGF	0.677	growth factor		1.203	3.61E-12
FGF2	-3.79	growth factor		0.195	3.65E-12
CBX5	-0.136	transcription regulator		0.973	4.29E-12
TWIST1	-1.505	transcription regulator	Inhibited	-3.452	7.13E-12

Supplementary Table 13. Reagents and resources used and generated in the study.

REAGENT or RESOURCE	SOURCE	IDENTIFIER
**Antibodies**		
Rabbit polyclonal anti-AR	Millipore	06-680
Rabbit monoclonal anti-β-Catenin	Cell Signaling Technology	8480
Rabbit monoclonal anti-SOX2	Cell Signaling Technology	14962
Rabbit monoclonal anti-COX2	Cell Signaling Technology	12282
Rabbit monoclonal anti-Cyclin D1	Cell Signaling Technology	2978
Rabbit monoclonal anti-phospho-Rb (Ser780)	Cell Signaling Technology	8180
Rabbit monoclonal anti-GAPDH	Cell Signaling Technology	5174
Rabbit monoclonal anti-Ki67	ThermoFisher	RM9106S1
Rabbit polyclonal anti-Ki67	abcam	ab833
Rabbit monoclonal anti-Cleaved Caspase-3 (Asp175)	Cell Signaling Technology	9661
Rat monoclonal anti-Mouse CD16/CD32 (Mouse Fc Block), Clone 2.4G2	BD Biosciences	553141
CD3e Monoclonal Antibody (145-2C11), Functional Grade	eBioscience	16-0031
CD28 Monoclonal Antibody (37.51), Functional Grade	eBioscience	16-0281
Rabbit monoclonal anti-CD11b	abcam	ab133357
Rabbit monoclonal anti-Ly6G (clone 1A8)	BioLegend	127602
Rabbit monoclonal anti-FoxP3	Cell Signaling Technology	12653
Rabbit monoclonal anti-p-Akt (Ser473)	Cell Signaling Technology	4060
Mouse monoclonal anti-Akt (pan)	Cell Signaling Technology	2920
Rabbit monoclonal anti-FoxP3	Cell Signaling Technology	12653
Goat polyclonal anti-CDH3	R&D Systems	AF761
Rabbit polyclonal anti-p16^{INK4a}	Santa Cruz Biotechnology	sc-1207
Rabbit polyclonal anti-p19^{ARF}	abcam	ab80
Rabbit polyclonal anti-p-HER2 (Tyr1248)	Cell Signaling Technology	2247
Rabbit monoclonal anti-p-HER2 (Tyr1221/1222)	Cell Signalling Technology	2243
Rabbit monoclonal anti-HER2	Cell Signalling Technology	2165
Rabbit polyclonal anti-CD3e	DAKO	A0452
Mouse monoclonal anti-CD8a (clone C8/144B)	BioLegend	372902
Mouse monoclonal anti-CD68 (clone KP1)	ThermoFisher	MS397P
Rabbit monoclonal anti-PD-L1	Cell Signaling Technology	13684
Rabbit polyclonal anti-cytokeratin 5	Biolegend	PRB-160P
Rabbit monoclonal anti-SMAD4	Cell Signaling Technology	46535
Rabbit polyclonal anti-APC	Santa Cruz Biotechnology	sc-896
Rabbit monoclonal anti-PTEN	Cell Signaling Technology	9559
Mouse monoclonal β-actin	Santa Cruz Biotechnology	sc-47778
139La, anti-FAK, clone D2R2E	Cell Signaling Technology	13009BF
141Pr, anti-Gr-1, clone RB6-8C5	Fluidigm	3141005B
142Nd, anti-CD11c, Polyclonal	Fluidigm	3142003B
143Nd, anti-IL-5, clone TRFK5	Fluidigm	3143003B
144Nd, anti-IL-2, clone JES6-5H4	Fluidigm	3144002B
145Nd, anti-CD69, clone H1.2F3	Fluidigm	3145005B
146Nd, anti-CD8a, clone 53-6.7	Fluidigm	3146003B
147Sm, anti-CD45, clone 30-F11	Fluidigm	3147003B
148Nd, anti-CD11b, clone M1/70	Fluidigm	3148003B
149Sm, anti-CD19, clone 6D5	Fluidigm	3149002B
150Nd, anti-CD25, clone 3C7	Fluidigm	3150002B
151Eu, anti-CD133, clone 315-2C11	BioLegend	141202

152Sm, anti-CD3e, clone 145-2C11	Fluidigm	3152004B
153Eu, anti-p-JNK, p-SAPK, p-MAPK8/9, clone N9-66	BD Biosciences	562480
154Sm, anti-LKB1, clone D60C5	Cell Signaling Technology	3047BF
155Gd, anti-ICOS, clone C398.4A	BioLegend	313502
156Gd, anti-CD34, clone MEC14.7	BioLegend	119302
158Gd, anti-Foxp3, clone FJK-16s	Fluidigm	3158003A
159Tb, anti-p-AKT, clone M89-61	BD Biosciences	560397
160Gd, anti-CD62L, clone MEL-14	Fluidigm	3160008B
161Dy, anti-PD-1, clone RMP1-14	BioLegend	114102
162Dy, anti-Ter119, clone TER-119	Fluidigm	3162003B
163Dy, anti-CTLA-4, clone 9H10	BioLegend	106202
164Dy, anti-p-mTOR, clone D9C2	Cell Signaling Technology	5536BF
165Ho, anti-CD31, clone 390	Fluidigm	3165013B
166Er, anti-IL-4, clone 11B11	Fluidigm	3166003B
167Er, anti-IL-6, clone MP5-20F3	Fluidigm	3167003B
169Tm, anti-TCRbeta, clone H57-597	Fluidigm	3169002B
170Er, anti-NK1.1, clone PK136	Fluidigm	3170002B
171Yb, anti-CD44, clone IM7	Fluidigm	3171003B
172Yb, anti-CD4, clone RM4-5	Fluidigm	3172003B
173Yb, anti-F4/80, clone BM8	BioLegend	123102
174Yb, anti-CD326, clone G8.8	BioLegend	118201
175Lu, anti-p-S6, clone N7-548	Fluidigm	3175009A
176Yb, anti-B220, clone RA3-6B2	Fluidigm	3176002B
**Biological Samples**		
Achieved formalin-fixed paraffin-embedded PSCC samples	MD Anderson Cancer Center	https://www.mdanderson.org/
Penis disease spectrum tissue array, including TNM, clinical stage and pathology grade, N=208	US Biomax, Inc.	PE2081
Penis cancer tissue array with unmatched normal adjacent tissues, N=24	US Biomax, Inc.	PE241
**Chemicals, Peptides, and Recombinant Proteins**		
5-Azacytidine	Cayman	11164
Decitabine	Cayman	11166-5
Erlotinib	Cayman	10483
Mubritinib	Cayman	12096
Palbociclib	ChemieTek	CT-PD2991
Dorsomorphin	LC Laboratories	D-3197
Everolimus	LC Laboratories	E-4040
GDC-0941	LC Laboratories	G-9252
Paclitaxel	LC Laboratories	P-9600
Rapamycin	LC Laboratories	R-5000
Ruxolitinib	LC Laboratories	R-6600
Temsirolimus	LC Laboratories	T-8040
Lapatinib	LC labs	L-4804
BP-1-102	Millipore	573132
LLL12	Millipore	573131
S3I-201	Millipore	573102
UO126	Millipore	662005
MK-2206	Selleck Chemicals	S1078
PD325901	Selleck Chemicals	S1036
PFI-1	Selleck Chemicals	S1216
Ridaforolimus	Selleck Chemicals	S1022

RO4929097	Selleck Chemicals	S1575
MK-8776 (SCH 900776)	Selleck Chemicals	S2735
Staurosporine	Selleck Chemicals	S1421
Trichostatin A	Selleck Chemicals	S1045
WP1066	Selleck Chemicals	S2796
Navitoclax (ABT-263)	Selleck Chemicals	S1001
Vistusertib (AZD2014)	Selleck Chemicals	S2783
AZD7762	Selleck Chemicals	S1532
AZD8055	Selleck Chemicals	S1555
Dactolisib (BEZ235)	Selleck Chemicals	S1009
BMS-754807	Selleck Chemicals	S1124
BMS-911543	Selleck Chemicals	S7144
Cabozantinib	Selleck Chemicals	S1119
Dasatinib	Selleck Chemicals	S1021
Enzalutamide	Selleck Chemicals	S1250
Ifosfamide	Selleck Chemicals	S1302
Cisplatin	Sigma Aldrich	P4394
(+)-JQ1	TOCRIS	4499
ABT-737	Toronto Research Chemicals	A112550
Buparlisib (BKM120)	Toronto Research Chemicals	N925760
PF-4708671	Toronto Research Chemicals	P294010
Celecoxib	LC Laboratories	C-1502
**Critical Commercial Assays**		
Human Tumor Dissociation Kit	Miltenyi Biotec	130-095-929
Mouse Tumor Dissociation Kit	Miltenyi Biotec	130-096-730
Myeloid-Derived Suppressor Cell Isolation Kit, mouse	Miltenyi Biotec	130-094-538
Pan T Cell Isolation Kit II, mouse	Miltenyi Biotec	130-095-130
MycoAlert Mycoplasma Detection Kit	Lonza	LT07-318
Cell-ID Cisplatin-195Pt	Fluidigm	201064
MaxPar Nucleic Acid Intercalator-Ir	Fluidigm	201192B
Foxp3 / Transcription Factor Staining Buffer Set	eBioscience	00-5523-00
Vybrant CFDA SE Cell Tracer Kit	Invitrogen	V12883
**Deposited Data**		
Raw Data Files from RNA-seq	This paper	GEO: GSE130052
**Experimental Models: Cell Lines**		
Mouse: PSCC cell line SA1	This paper	N/A
Mouse: PSCC cell line SAP1	This paper	N/A
Mouse: PSCC cell line SA1_shControl	This paper	N/A
Mouse: PSCC cell line SA1_shSox2	This paper	N/A
**Experimental Models: Organisms/Strains**		
Mouse: PB-Cre4^{+} Smad4$^{L/L}$ Apc$^{L/L}$ (SA)	This paper	N/A
Mouse: PB-Cre4^{+} Smad4$^{L/L}$ Apc$^{L/L}$ Pten$^{L/L}$ (SAP)	This paper	N/A
Mouse: C57BL/6J	Jackson Laboratory	000664
Mouse: C.129S7(B6)-Rag1^{tm1Mom}/J (Rag1 KO)	Jackson Laboratory	003145
**Recombinant DNA**		
shRNA sequence targeting 3UTR of mouse Sox2: CCGGCAAAGAGATACAAGGGAATTGCTCGAGCAATTCCCTTGTATCTCTTTGTTTTTG	Sigma-Aldrich	SHCLNG-NM_011443 (TRCN0000416106)
pLKO.1-puro Empty Vector Control	Sigma-Aldrich	SHC001

**Software and Algorithms**		
Prism version 7	Graphpad	https://www.graphpad.com/scientific-software/prism/
Cytobank	Cytobank	https://www.cytobank.org/
Ingenuity Pathway Analysis	QIAGEN Bioinformatics	https://www.qiagenbioinformatics.com/products/ingenuity-pathway-analysis/
**Other**		
BD LSRFortessa Cell Analyzer	BD Biosciences	N/A
Epoch 2 Microplate Spectrophotometer	BioTek	N/A
