# Supplementary material for: Effective combinatorial immunotherapy for penile squamous cell carcinoma
Source: Nat Commun. 2020 May 1;11:2124. doi: 10.1038/s41467-020-15980-9 (PMC7195486; doi:10.1038/s41467-020-15980-9)
Supplement: Supplementary file 3 — Reporting Summary [file 41467_2020_15980_MOESM3_ESM.pdf]

nature research

Corresponding author(s): Xin Lu

Last updated by author(s): Apr 6, 2020

Reporting Summary

Nature Research wishes to improve the reproducibility of the work that we publish. This form provides structure for consistency and transparency in reporting. For further information on Nature Research policies, see Authors & Referees and the Editorial Policy Checklist.

Statistics

For all statistical analyses, confirm that the following items are present in the figure legend, table legend, main text, or Methods section.

n/a	Confirmed	
☐	☒	The exact sample size (*n*) for each experimental group/condition, given as a discrete number and unit of measurement
☐	☒	A statement on whether measurements were taken from distinct samples or whether the same sample was measured repeatedly
☐	☒	The statistical test(s) used AND whether they are one- or two-sided<br>*Only common tests should be described solely by name; describe more complex techniques in the Methods section.*
☒	☐	A description of all covariates tested
☐	☒	A description of any assumptions or corrections, such as tests of normality and adjustment for multiple comparisons
☐	☒	A full description of the statistical parameters including central tendency (e.g. means) or other basic estimates (e.g. regression coefficient) AND variation (e.g. standard deviation) or associated estimates of uncertainty (e.g. confidence intervals)
☐	☒	For null hypothesis testing, the test statistic (e.g. *F*, *t*, *r*) with confidence intervals, effect sizes, degrees of freedom and *P* value noted<br>*Give P values as exact values whenever suitable.*
☒	☐	For Bayesian analysis, information on the choice of priors and Markov chain Monte Carlo settings
☒	☐	For hierarchical and complex designs, identification of the appropriate level for tests and full reporting of outcomes
☒	☐	Estimates of effect sizes (e.g. Cohen's *d*, Pearson's *r*), indicating how they were calculated

Our web collection on statistics for biologists contains articles on many of the points above.

Software and code

Policy information about availability of computer code

Data collection	No software was used.
Data analysis	Graphpad Prism version 7, Cytobank 6.3.1, R version 3.3.3, and Ingenuity Pathway Analysis (QIAGEN)

For manuscripts utilizing custom algorithms or software that are central to the research but not yet described in published literature, software must be made available to editors/reviewers. We strongly encourage code deposition in a community repository (e.g. GitHub). See the Nature Research guidelines for submitting code & software for further information.

Data

Policy information about availability of data

All manuscripts must include a data availability statement. This statement should provide the following information, where applicable:

- Accession codes, unique identifiers, or web links for publicly available datasets
- A list of figures that have associated raw data
- A description of any restrictions on data availability

The RNAseq data generated in the study have been deposited and available for public access in the Gene Expression Omnibus (GEO) database under the accession code GSE130052 [https://www.ncbi.nlm.nih.gov/geo/query/acc.cgi?acc=GSE130052]. The GSE57955 [https://www.ncbi.nlm.nih.gov/geo/query/acc.cgi?acc=GSE57955] data referenced during the study are available in a public repository from the GEO website. All the other data supporting the findings of this study are available within the article and its supplementary information files and from the corresponding author upon reasonable request. A reporting summary for this article is available as a Supplementary Information file.

Field-specific reporting

Please select the one below that is the best fit for your research. If you are not sure, read the appropriate sections before making your selection.

☒ Life sciences ☐ Behavioural & social sciences ☐ Ecological, evolutionary & environmental sciences

For a reference copy of the document with all sections, see nature.com/documents/nr-reporting-summary-flat.pdf

Life sciences study design

All studies must disclose on these points even when the disclosure is negative.

Sample size	Sample size was chosen based on our previous experience with similar type of experiments (PMID: 26701088; PMID: 28321130;). Calculations were based on expected standard deviation and mean in groups and expected alpha 0.05 with power 0.8.
Data exclusions	No data was excluded for in vitro experiments. For animal experiments, if a mouse died due to other condition than tumor burden, it was excluded from the analysis.
Replication	As reported in the figure legends and the Statistics and reproducibility section of Methods, the findings were generated with sufficient biological replicates.
Randomization	For all in vivo experiments, animals were randomly assigned into a treatment group after detection of tumor formation. The starting tumor burden in the treatment and control groups was similar before treatment. For in vitro experiments such as western blot and immunohistochemistry, samples are allocated to experimental groups based on the nature of the samples. Samples of the same group were randomized during experiments as much as possible, e.g. the order of loading to the gel, the order of staining, the order of mcroscopy.
Blinding	Blinding was not always possible for animal experiments, due to the fact that the main investigators were aware of the design. However, when technicians performed the drug treatment experiments in animal studies, blinding was practiced. This same principle applies to experiments other than animal experiments. For example, the technician was given samples to run western blot with only sample IDs but not the information on the animal groups which the samples were extracted from.

Reporting for specific materials, systems and methods

We require information from authors about some types of materials, experimental systems and methods used in many studies. Here, indicate whether each material, system or method listed is relevant to your study. If you are not sure if a list item applies to your research, read the appropriate section before selecting a response.

Materials & experimental systems

n/a	Involved in the study	
☐	☒	Antibodies
☐	☒	Eukaryotic cell lines
☒	☐	Palaeontology
☐	☒	Animals and other organisms
☐	☒	Human research participants
☒	☐	Clinical data

Methods

n/a	Involved in the study	
☒	☐	ChIP-seq
☒	☐	Flow cytometry
☒	☐	MRI-based neuroimaging

Antibodies

Antibodies used

The list of antibodies used can be found in Supplementary Table 13. Also listed below:
Rabbit polyclonal anti-AR Millipore 06-680
Rabbit monoclonal anti-β-Catenin Cell Signaling Technology 8480
Rabbit monoclonal anti-SOX2 Cell Signaling Technology 14962
Rabbit monoclonal anti-COX2 Cell Signaling Technology 12282
Rabbit monoclonal anti-Cyclin D1 Cell Signaling Technology 2978
Rabbit monoclonal anti-phospho-Rb (Ser780) Cell Signaling Technology 8180
Rabbit monoclonal anti-GAPDH Cell Signaling Technology 5174
Rabbit monoclonal anti-Ki67 ThermoFisher RM9106S1
Rabbit polyclonal anti-Ki67 abcam ab833
Rabbit monoclonal anti-Cleaved Caspase-3 (Asp175) Cell Signaling Technology 9661
Rat monoclonal anti-Mouse CD16/CD32 (Mouse Fc Block), Clone 2.4G2 BD Biosciences 553141
CD3e Monoclonal Antibody (145-2C11), Functional Grade eBioscience 16-0031
CD28 Monoclonal Antibody (37.51), Functional Grade eBioscience 16-0281
Rabbit monoclonal anti-CD11b abcam ab133357
Rabbit monoclonal anti-Ly6G (clone 1A8) BioLegend 127602
Rabbit monoclonal anti-FoxP3 Cell Signaling Technology 12653

Rabbit monoclonal anti-p-Akt (Ser473) Cell Signaling Technology 4060
Mouse monoclonal anti-Akt (pan) Cell Signaling Technology 2920
Rabbit monoclonal anti-FoxP3 Cell Signaling Technology 12653
Goat polyclonal anti-CDH3 R&D Systems AF761
Rabbit polyclonal anti-p16INK4a Santa Cruz Biotechnology sc-1207
Rabbit polyclonal anti-p19ARF abcam ab80
Rabbit polyclonal anti-p-HER2 (Tyr1248) Cell Signaling Technology 2247
Rabbit monoclonal anti-p-HER2 (Tyr1221/1222) Cell Signalling Technology 2243
Rabbit monoclonal anti-HER2 Cell Signalling Technology 2165
Rabbit polyclonal anti-CD3e DAKO A0452
Mouse monoclonal anti-CD8a (clone C8/144B) BioLegend 372902
Mouse monoclonal anti-CD68 (clone KP1) ThermoFisher MS397P
Rabbit monoclonal anti-PD-L1 Cell Signaling Technology 13684
Rabbit polyclonal anti-cytokeratin 5 Biolegend PRB-160P
Rabbit monoclonal anti-SMAD4 Cell Signaling Technology 46535
Rabbit polyclonal anti-APC Santa Cruz Biotechnology sc-896
Rabbit monoclonal anti-PTEN Cell Signaling Technology 9559
Mouse monoclonal β-actin Santa Cruz Biotechnology sc-47778
139La, anti-FAK, clone D2R2E Cell Signaling Technology 13009BF
141Pr, anti-Gr-1, clone RB6-8C5 Fluidigm 3141005B
142Nd, anti-CD11c, Polyclonal Fluidigm 3142003B
143Nd, anti-IL-5, clone TRFK5 Fluidigm 3143003B
144Nd, anti-IL-2, clone JES6-5H4 Fluidigm 3144002B
145Nd, anti-CD69, clone H1.2F3 Fluidigm 3145005B
146Nd, anti-CD8a, clone 53-6.7 Fluidigm 3146003B
147Sm, anti-CD45, clone 30-F11 Fluidigm 3147003B
148Nd, anti-CD11b, clone M1/70 Fluidigm 3148003B
149Sm, anti-CD19, clone 6D5 Fluidigm 3149002B
150Nd, anti-CD25, clone 3C7 Fluidigm 3150002B
151Eu, anti-CD133, clone 315-2C11 BioLegend 141202
152Sm, anti-CD3e, clone 145-2C11 Fluidigm 3152004B
153Eu, anti-p-JNK, p-SAPK, p-MAPK8/9, clone N9-66 BD Biosciences 562480
154Sm, anti-LKB1, clone D60C5 Cell Signaling Technology 3047BF
155Gd, anti-ICOS, clone C398.4A BioLegend 313502
156Gd, anti-CD34, clone MEC14.7 BioLegend 119302
158Gd, anti-Foxp3, clone FJK-16s Fluidigm 3158003A
159Tb, anti-p-AKT, clone M89-61 BD Biosciences 560397
160Gd, anti-CD62L, clone MEL-14 Fluidigm 3160008B
161Dy, anti-PD-1, clone RMP1-14 BioLegend 114102
162Dy, anti-Ter119, clone TER-119 Fluidigm 3162003B
163Dy, anti-CTLA-4, clone 9H10 BioLegend 106202
164Dy, anti-p-mTOR, clone D9C2 Cell Signaling Technology 5536BF
165Ho, anti-CD31, clone 390 Fluidigm 3165013B
166Er, anti-IL-4, clone 11B11 Fluidigm 3166003B
167Er, anti-IL-6, clone MP5-20F3 Fluidigm 3167003B
169Tm, anti-TCRbeta, clone H57-597 Fluidigm 3169002B
170Er, anti-NK1.1, clone PK136 Fluidigm 3170002B
171Yb, anti-CD44, clone IM7 Fluidigm 3171003B
172Yb, anti-CD4, clone RM4-5 Fluidigm 3172003B
173Yb, anti-F4/80, clone BM8 BioLegend 123102
174Yb, anti-CD326, clone G8.8 BioLegend 118201
175Lu, anti-p-S6, clone N7-548 Fluidigm 3175009A
176Yb, anti-B220, clone RA3-6B2 Fluidigm 3176002B

Validation

There are two sources of validation.
1) For CyTOF antibodies, we validated their use in our previous publication (PMID: 26701088). These antibodies were provided to us as part of the facility service from the antibody bank of the Flow Cytometry and Cellular Imaging Core Facility at MD Anderson Cancer Center (https://www.mdanderson.org/research/research-resources/core-facilities/flow-cytometry-and-cellular-imaging-core-facility.html). They have independent validation for their bank antibodies.
2) For the other antibodies, we purchased them from reputable commercial vendors (e.g. CST, Abcam) based on either prior literature use of the antibodies or the validation information associated with the datasheet. We typically identified which antibodies to purchase based on the highest independent usage reports in the literature using the online antibody search engine benchsci.com

Eukaryotic cell lines

Policy information about cell lines

Cell line source(s)

Murine penile cancer cell line SA1 and SAP1 were derived from established penile tumors of SA and SAP mice, respectively. Lentivirus packaging cell line 293T was purchased originally from ATCC (CRL-3216).

Authentication

These newly established murine cell lines were cultured independently of any other cell cultures. They were not authenticated. The lentivirus packaging cell line 293T was a tool cell line, and it was not subject to further authentication

	procedure after the initial purchase, although we constantly monitored and ensured that the morphology of the cells under microscope was consistent with ATCC posted image (https://www.atcc.org/~/media/Attachments/3/1/D/7/CRL-3216%20Low.ashx).
Mycoplasma contamination	All cell lines in our laboratory are routinely tested for mycoplasma contamination using PCR-based methods and cells used in this study were negative for mycoplasma.
Commonly misidentified lines (See ICLAC register)	No cell line used in the paper is listed in ICLAC database.

Animals and other organisms

Policy information about studies involving animals; ARRIVE guidelines recommended for reporting animal research

Laboratory animals	C57BL/6 males and Rag1 knockout males at 6 weeks old were purchased from Jackson Laboratories (stock# 000664 and 003145, respectively). NCr nude males at 6 weeks old were purchased from Taconic (NCRNU-M).Genetic alleles PB-Cre4, PtenL/L, Smad4L/L, mTmGL/L and ApcL/L were recently described in our studies. These alleles were bred to form SA and SAP colonies, from which males of the SA or SAP genotype and indicated ages in the figures and figure legends were used for experiments. The mice were maintained in 12hr/12hr light/dark with an ambient temperature of 72F and humidity of 35%.
Wild animals	Not used
Field-collected samples	Not used
Ethics oversight	All animal works performed in this study were approved by The University of Texas MD Anderson Cancer Center Institutional Animal Care and Use Committee and The University of Notre Dame Institutional Animal Care and Use Committee. All animals were maintained in pathogen-free conditions and cared for in accordance with the International Association for Assessment and Accreditation of Laboratory Animal Care (AAALAC) policies and certification.

Note that full information on the approval of the study protocol must also be provided in the manuscript.

Human research participants

Policy information about studies involving human research participants

Population characteristics	The eight patient specimen cohort (see Supplementary Table 1) was retrieved from banked archival tissue specimens among patients who had previously consented to have their incidental specimens banked for future research at the University of Texas M.D. Anderson Cancer Center utilizing an approved protocol. Specific blocks were selected based upon the abundance of tissue for additional studies (i.e., usually tumors greater than 1 cm in size) and the patients were further characterized with respect to age, clinical stage, grade, histology, type of surgery, and Human papillomavirus status.
Recruitment	Specific blocks were selected based upon the abundance of tissue for additional studies (i.e., usually tumors greater than 1 cm in size) and the patients were further characterized with respect to age, clinical stage, grade, histology, type of surgery, and Human papillomavirus status. There was no bias in terms of selecting the samples that is expected to impact the results presented in the manuscript.
Ethics oversight	De-identified specimens were provided to the research team under an institutional protocol (PA15-0138) for further characterization under waiver of consent.

Note that full information on the approval of the study protocol must also be provided in the manuscript.
